# BIPSPI: a method for the prediction of partner-specific protein–protein interfaces

**DOI:** 10.1093/bioinformatics/bty647

**Published:** 2018-07-18

**Authors:** Ruben Sanchez-Garcia, C O S Sorzano, J M Carazo, Joan Segura

**Affiliations:** GN7 of the Spanish National Institute for Bioinformatics (INB), Biocomputing Unit, National Center of Biotechnology (CSIC), Instruct Image Processing Center, Madrid, Spain

## Abstract

**Motivation:**

Protein–Protein Interactions (PPI) are essentials for most cellular processes and thus, unveiling how proteins interact is a crucial question that can be better understood by identifying which residues are responsible for the interaction. Computational approaches are orders of magnitude cheaper and faster than experimental ones, leading to proliferation of multiple methods aimed to predict which residues belong to the interface of an interaction.

**Results:**

We present BIPSPI, a new machine learning-based method for the prediction of partner-specific PPI sites. Contrary to most binding site prediction methods, the proposed approach takes into account a pair of interacting proteins rather than a single one in order to predict partner-specific binding sites. BIPSPI has been trained employing sequence-based and structural features from both protein partners of each complex compiled in the Protein–Protein Docking Benchmark version 5.0 and in an additional set independently compiled. Also, a version trained only on sequences has been developed. The performance of our approach has been assessed by a leave-one-out cross-validation over different benchmarks, outperforming state-of-the-art methods.

**Availability and implementation:**

BIPSPI web server is freely available at http://bipspi.cnb.csic.es. BIPSPI code is available at https://github.com/bioinsilico/BIPSPI. Docker image is available at https://hub.docker.com/r/bioinsilico/bipspi/.

**Supplementary information:**

Supplementary data are available at *Bioinformatics* online.

## 1 Introduction

Protein–Protein Interactions (PPI’s) are at the basis of virtually every cellular process. Therefore, elucidating the biochemical underpinnings of interactions is a fundamental step for improving our understanding of cellular mechanisms and diseases. Much research has been done on PPI’s, especially at cellular level, which has led to the availability of many interactomes (Cafarelli *et al.*, 2017). However, in order to grasp protein function in cellular processes, not only it is important to know which proteins interact but how proteins bind to their different partners and thus, identifying protein–protein interfaces becomes a central issue.

Many experimental methodologies exist for the characterization of protein–protein interfaces, including mass spectrometry (Sobott and Robinson, 2002), mutagenesis (Chen *et al.*, 2014), X-ray crystallography (Shi, 2014) or nuclear magnetic resonance (O'Connell *et al.*, 2009). Nevertheless, in many cases, these approaches require expensive and time-consuming experiments and are not suitable for the analysis of large datasets. As a result, many computational approaches have been designed to predict and characterize PPI’s at different levels. For example, several protein–protein docking approaches (Rodrigues *et al.*, 2015; Zhang *et al.*, 2016) have been developed to obtain atomic models for the interaction of two proteins when solved structures of both partners are available. For those other cases in which there is no structural information, or it only exists at low resolution, other methods to identify which pairs of domains are likely to bind in PPI’s have been proposed (Segura *et al.*, 2015b; Segura *et al.*, 2016; Wang *et al.*, 2007). Nonetheless, most approaches work at residue level predicting which protein residues constitute binding sites or interfaces of a protein complex. Generally, these algorithms employ knowledge derived from structurally solved protein in order to build templates or statistical models (Xue *et al.*, 2015).

Different knowledge-based methods can be found in the scientific literature. Some of them use homology information, inferring protein interfaces from templates of homologous complexes (Mosca *et al.*, 2013; Segura *et al.*, 2016; Xue *et al.*, 2011). Other approaches employ correlated mutations in order to identify pairs of residues that are likely to interact (Jones *et al.*, 2012; Morcos *et al.*, 2014; Pazos *et al.*, 1997). On the other hand, most proposed data-driven methods make use of machine learning algorithms that are trained on heterogeneous sets of structurally solved complexes (Ahmad and Mizuguchi, 2011; de Vries *et al.*, 2006; Fout *et al.*, 2017; Hwang *et al.*, 2016; Meyer *et al.*, 2018; Minhas *et al.*, 2014; Murakami and Mizuguchi, 2010; Neuvirth *et al.*, 2004; Porollo and Meller, 2006; Savojardo *et al.*, 2017; Segura *et al.*, 2011, 2012). The different strategies have different relative merits, depending on the context. For example, template-based approaches might offer accurate predictions when homologue complexes are available (Xue *et al.*, 2015). Similarly, correlated mutations have been shown to provide very useful information when high quality multiple sequence alignments can be compiled (Ovchinnikov *et al.*, 2014). On the other hand, machine-learning solutions are not limited by the need of high quality templates or alignments, so that they can be used in more general contexts. Finally, docking algorithms, which are able to achieve atomic resolution in their prediction but are also computationally demanding, can benefit from data-driven predictions in order to get faster and more accurate solutions (Rodrigues *et al.*, 2015; Segura *et al.*, 2015a).

Several formulations can be found to the problem of predicting protein complex interfaces or binding sites (Ahmad and Mizuguchi, 2011). On the one hand, partner-independent binding site predictions aim to identify all residues of a given protein that interact with any protein. On the other hand, partner-specific binding site predictions (from now on ‘interface predictions’) pursue to identify which residues are involved in a particular PPI. Partner-specificity is a desirable attribute for interface predictors as most proteins interact with several partners (Grigoriev, 2003) and the interfaces for each partner can be totally different. This is especially true for transient interactions, which are fundamental for processes such as signal transduction (Xue *et al.*, 2015). It is not then surprising that partner-specific methods tend to outperform non-specific approaches (Ahmad and Mizuguchi, 2011; Minhas *et al.*, 2014). However, most current binding site prediction approaches based on machine learning algorithms are designed to achieve non-partner specific predictions. Indeed, to our knowledge, only a few machine-learning based methods computing partner-specific binding sites are currently available. Ahmad *et al.* proposed PPiPP, an ensemble of 24 neural networks which used amino acid type and PSSMs (Position Specific Scoring Matrices) through a sliding window as features to predict binding sites on protein sequences (Ahmad and Mizuguchi, 2011). PAIRpred (Minhas *et al.*, 2014), one of the state-of-the-art methods, is a Support Vector Machine that employs a specific pairwise kernel over a set of structural and/or sequence-based features. This latter set of sequence-based features is comprised by PSSMs, PSFMs (Position Specific Frequency Matrices) and solvent accessibility predictions, while the structural descriptors include residue depth, solvent accessibility, protrusion index and half sphere amino acid compositions. In general, better performance is achieved when structures of the protein partners are available. Recently, Fout *et al.* developed a graph convolutional neural network (GCNN) method using the set of features described in PAIRpred (Fout *et al.*, 2017). Finally, the ECLAIR method (Meyer *et al.*, 2018), which was designed to function in high-throughput scenarios, is based on an ensemble of Random Forests, each of them trained on a different set of features including biophysical, structure-base, docking-based and co-evolution features.

In this work, we present BIPSPI (xgBoost Interface Prediction of Specific-Partner Interactions), a new machine-learning based method for the partner-specific prediction of residue–residue contacts and binding sites. BIPSPI can predict interface residues from either protein sequences or protein structures. To that end, BIPSPI employs multiple structural and/or sequence-based amino acid features that are combined through an Extreme Gradient Boosting (XGBoost) (Chen and Guestrin, 2016) model and a new scoring function that converts residue contact predictions into binding site scores. BIPSPI performance has been evaluated by means of a leave-one-out cross-validation over different datasets (Hwang *et al.*, 2008; Vreven *et al.*, 2015) and against an independent testing set derived from CAPRI targets (Janin *et al.*, 2003). Finally, BIPSPI was compared with similar methods, outperforming previous reported results. A web server where BIPSPI can be employed and results and datasets downloaded is freely available at http://bipspi.cnb.csic.es.

## 2 Materials and methods

BIPSPI classifier has been trained to predict interfaces from protein structures and/or sequences. This section describes the implementation of the method using structural data. A full description of the sequence-based classifier is available in Supplementary Section S1.

### 2.1 Datasets

Different sets of protein complexes were used to train and evaluate the performance of BIPSPI predicting protein interfaces. The first one was the Protein–Protein Docking Benchmark version 5 (Vreven *et al.*, 2015) dataset, that contains 230 non-redundant protein complexes for which bound and unbound structures are available. Each of these complexes has resolution better than 3.25 Å and the length of each sequence is >30 amino acids. To avoid redundancy, this dataset was compiled ensuring that none of the protein complexes belonged to the same pair of SCOP families (Andreeva *et al.*, 2008). This set will be referred to as DBv5. The second dataset was the Protein–Protein Docking Benchmark version 3, in this work termed DBv3, (Hwang *et al.*, 2008), which is a subset of DBv5.

In addition to DBv5 and DBv3, we have compiled a new dataset of 117 protein dimers (DImS) following a similar approach as the one used to compile the different Protein–Protein Docking Benchmark versions. This dataset was built selecting PDB dimers of at least 35 amino acids long, with resolutions better than 3 Å and for which >90% of their residues were structurally determined. Similar to DBv5 and DBv3, non-redundancy between protein complexes was established at SCOP family level in such a way that only dimers with one SCOP domain per partner were considered and none of the dimers shared the same combination of SCOP families (see Supplementary Section S6.12 for a detailed list). Finally, several CAPRI targets (see Supplementary Section S6.12 for a detailed list) were also employed as independent testing data and as a way to provide a direct comparison with other methods (Savojardo *et al.*, 2017).

### 2.2 Residue–residue contact definition

Different definitions of residue–residue contact can be found in the scientific literature (Xue *et al.*, 2015). Two of the most commonly used are: (i) residue solvent accessibility reduction after complex formation and (ii) distance threshold between residue heavy atoms. In order to compare with other existent methods, we have adopted the same contact definition that was used in PPiPP (Ahmad and Mizuguchi, 2011) and PAIRpred (Minhas *et al.*, 2014). Accordingly, a pair of residues is defined as interacting if the distance between any of their heavy atoms is <6.0 Å.

In our analysis, we found that in DBv5 there are 20 799 interacting residue pairs as opposed to 15 333 317 non-interacting residue pairs; thus, an extreme class imbalance. To properly handle this situation during the different training steps of BIPSPI, we only considered random samples of all non-interacting pairs, including non-accessible residues to account for possible conformational changes. Several sampling proportions were tested, achieving the best performance when the number of selected negative cases was three times larger than the number of interacting pairs (data not shown). This random sample, which also makes training faster, is drawn at protein complex level, in such a way that all complexes contribute to the dataset with the same relation of positive and negative cases. Finally, it is important to notice that no sampling is done for evaluation and, hence, it is performed on whole proteins data.

### 2.3 Data encoding

Residue pairs are codified as a vector of numerical features. In this work, a protein A is defined as a collection of residues $\alpha_{i}$ and a pair of residues $\left(\alpha_{i},\beta_{j}\right)$ will identify two amino acids belonging to proteins A and B, respectively. Due to the symmetry of the problem, each pair $\left(\alpha_{i},\beta_{j}\right)$ can also be defined as $\left(\beta_{j},\alpha_{i}\right)$. To tackle this, we have included both representations as different examples in the training set and, when computing scores, we have assigned the average of predictions for $\left(\alpha_{i},\beta_{j}\right)$ and predictions for $\left(\beta_{j},\alpha_{i}\right)$ to both of them. Next sections describe how the vector of features associated to a pair of residues $\left(\alpha_{i},\beta_{j}\right)$ is built.

#### 2.3.1 Single amino acid features

Each single residue is encoded by a set of sequence-based and structural features. Sequence-based features include amino acid type, codified as a vector of 22 binary elements (one-hot encoded), sequence profiles computed with PSI-BLAST (Altschul *et al.*, 1997) or retrieved from 3DCONS-DB (Sanchez-Garcia *et al.*, 2017) when available, and sequence conservation scores computed with AL2CO (Pei and Grishin, 2001). Structural features are also calculated to describe residues, including geometrical descriptors and hydrophobicity computed with PSAIA (Mihel *et al.*, 2008), one-hot encoded secondary structure determined by DSSP (Kabsch and Sander, 1983) and half-sphere exposure and contact number (Hamelryck, 2005) computed at radius of 12 Å with Biopython (Cock *et al.*, 2009). An exhaustive list of the residue features is available in Supplementary Section S2.

#### 2.3.2 Residue environment features

Residue environments are also described and included into the vector of features in such a way that for each feature its environment feature is calculated. Several residue environment definitions have been employed in different works, two of the most common ones are: (i) sequential environment through sliding window (Sikić *et al.*, 2009) and (ii) structural environment by Euclidean distance threshold (Porollo and Meller, 2006). In these works, three types of environments are used in combination: sequential environment, structural environment and structural pairwise environment.

Sequential environment is obtained by a sliding window approach of length 11 amino acids in which all sequence-based features described above are concatenated for all residues of the window. On the other hand, structural environment features are computed from all sequence-based and structural features of each residue employing a structural neighbourhood definition based on Voronoi Diagrams (Segura *et al.*, 2011). Basically, according to this definition, two residues are considered as neighbours if they share a common edge in the Voronoi Diagram defined by all C-α atoms of the protein. The computation of structural environment features is different depending whether the feature is represented by a real number or if it is represented as one-hot encoded. Hence, let $f_{i}$ be a real value feature for residue $\alpha_{i}$, then, its associated structural environment feature $ef_{i}$ is define as a set of four values:

(1) $$ef_{i}=\left\{\sum_{r\in N_{i}}f_{r},\frac{1}{|N_{i}|}\sum_{r\in N_{i}}f_{r}\underset{r\in N_{i}}{\max}f_{r},\underset{r\in N_{i}}{\min}f_{r}\right\}$$

Where $N_{i}$ is the set of neighbours of residue $\alpha_{i}$ according to Voronoi neighbourhood definition.

Similarly, let $h_{i}=(h_{i}^{1},h_{i}^{2},\ldots ,h_{i}^{k})$ be any one-hot encoded feature for

residue $\alpha_{i}$, where k is the number of classes of the feature h. Then, its associated structural environment feature of dimension k is computed as follows:

(2) $$eh_{i}=\sum_{r\in N_{i}}h_{r}$$

Residue pair scores predicted in the first step classifier are also included as new features in the second step (see Section 2.4); those scores can be regarded as pairwise features. Then, given a pair of residues $\left(\alpha_{i},\beta_{j}\right)$ and a pairwise score $F_{ij}$, the structural pairwise environment score $eF_{ij}$ is defined as:

(3) $$eF_{ij}=\left\{eF_{ij}^{\cdot j},eF_{ij}^{i\cdot },eF_{ij}^{\cdot \cdot }\right\}$$

where

(4) $$\;\;eF_{ij}^{\cdot j}=\left\{\sum_{r\in N_{i}}F_{rj},\frac{1}{|N_{i}|}\sum_{r\in N_{i}}F_{rj},\underset{r\in N_{i}}{\max}F_{rj},\underset{r\in N_{i}}{\min}F_{rj}\right\}$$

(5) $$eF_{ij}^{\cdot \cdot }=\left\{\sum_{r\in N_{i}}\sum_{s\in N_{j}}F_{rs},\frac{1}{|N_{i}||N_{j}|}\sum_{r\in N_{i}}\sum_{s\in N_{j}}F_{rs},\underset{r\in N_{i}s\in N_{j}}{\max}F_{rs},\underset{r\in N_{i}s\in N_{j}}{\min}F_{rs}\right\}$$

$N_{i}$ is the set of neighbours of residue $\alpha_{i}$ and $N_{j}$ is the set of neighbours of residue $\beta_{j}$  according to Voronoi neighbourhood definition.

### 2.4 BIPSPI algorithm

**Fig. 1.**
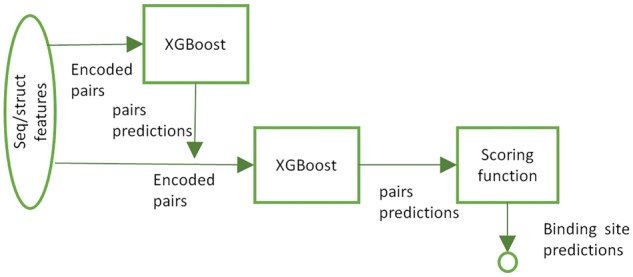
BIPSPI workflow. Sequence-base and structural features are used to codify pairs of residues. At first step, XGBoost classifier is fed with encoded pairs in order to obtain interacting pairs predictions. Interacting pairs scores are combined with original features and fed to a second step classifier. Lastly, interacting predictions obtained in step two are converted to binding site predictions employing our scoring function

BIPSPI algorithm was designed as a three steps workflow (see Fig. 1). First, a XGBoost classifier (Chen and Guestrin, 2016) is fed with the set of sequence-based and structural features and their respective environments. After that, a second XGBoost classifier is fed with the same input features adding the predictions that were obtained in the first step and their associated environment scores. Finally, a scoring function converts interacting pair predictions into binding site residue scores (see Section 2.5). The training procedure and selected algorithm hyperparameters are described in Supplementary Section S3.

### 2.5 From residue–residue contact scores to binding site prediction

In order to obtain individual interface residues, we have designed a scoring function to compute single amino acid binding site scores from residue–residue pairs results. This scoring function takes into account all residue pairs scores relying on the rank of the predictions when all pairs are sorted from highest to lowest score. Thus, the binding site score of a given residue is derived from the list of all pair predictions ordered from highest to lowest score using the following expression:

(6) $$I_{s}(\alpha )=\sum_{i\leq \log_{2}(n)}\frac{X_{c}(\alpha ,2^{i})}{2^{i}}$$

Where α is the particular residue whose score is computed, n is the number of residue pairs and $X_{c}(\alpha ,2^{i})$ is the number of times that residue α appears among the $2^{i}$ highest score pairs (see Supplementary Section S4). Additionally, with the aim of making predictions smoother, scores are averaged along the sequence employing a window size of three amino acids and using the vector of weights (1/4, 1/2, 1/4).

Finally, in order to provide a manageable score that allows for easy threshold selection, BIPSPI web server computes an expected precision value that is estimated using an isotonic regression model on the original scores (Zadrozny and Elkan, 2002) (see Supplementary Section S4.2 for more details).

### 2.6 Performance evaluation

The performance of BIPSPI predicting residue–residue contact pairs and binding sites was evaluated computing a leave-one-out cross-validation over the complexes included in the different datasets (see Section 2.1). Specifically, each of the classifiers of the method was trained with the sampled pairs of all protein complexes except for the ones that belong to the left-out complex. This evaluation procedure is the same that was used in PAIRpred and PPiPP and, when trained over DBv3, allows for a straight and fair comparison with those methods. In addition, several CAPRI targets interfaces were predicted as an independent benchmark. Residue–residue contact predictions (RRCP) were evaluated with the AUC values of ROC curves averaged over protein complexes (${\bar{AUC}}_{ROC}$) and mixing all residue–residue scores from the different complexes ($AUC_{ROC}$). Additionally, as these measurements can provide an over-optimistic view of performance due to the imbalance between interacting and non-interacting pairs, the AUC of the precision-recall curve ($AUC_{PR}$) is also provided. Single residue binding site predictions were also evaluated in terms of the Matthew Correlation Coefficient (MCC), precision (PR), recall (RC), specificity (SPC) and negative predictive value (NPV), which were computed at the threshold that maximized the MCC.

## 3 Results

### 3.1 BIPSPI feature importance analysis

The importance of the different features employed in BIPSPI has been analyzed by counting the total number of tree splits caused by each variable during model training (Friedman and Meulman, 2003). In order to obtain easily interpretable results, we have focused on families of features when those are classified by type (e.g. accessibility, conservation, secondary structure, etc.). Accordingly, the family of features with the highest contribution (sum of importance of all variables in the class), approximately 65%, was conservation. However, individually, the most informative variables belonged to the accessibility family for the first step and previous step prediction scores for the second one, being accessibility the next most important family feature. Additionally, we have studied the importance of the residue environment and observed that structural environment features explained >55% of the total importance despite being <31% of the total features number. An extended discussion of feature importance can be found in Supplementary Section S5.

### 3.2 BIPSPI performance analysis

The performance of BIPSPI predicting residue–residue contacts and binding sites was evaluated computing a leave-one-out cross-validation on DBv5 and DImS datasets. As expected, the method achieved the best performance when structural features and two classification steps were computed (see Table 1). Although the improvement in performance predicting residue–residue contacts between the first and second step is small, the improvement in performance predicting single residue binding sites after the second step is not negligible. For example, while BIPSPI ${\bar{AUC}}_{ROC}$ measured in DBv5 are 0.9011 and 0.9052 for the first and second step, respectively, the binding site ${\bar{AUC}}_{ROC}$ increases from 0.8046 in the first step to 0.8235 in the second one (see Table 1). This behaviour can be explained due to the high imbalance of interacting and non-interacting residue pairs, and, as a consequence, small improvements in residue–residue contact predictions can involve important improvements in binding site prediction.

**Table 1.** Performance evaluation for BIPSPI leave-one-out over the DBv5, DBv3 and DImS complexes and comparison with other methods

Algorithm	Dataset	Input	Residue–residue contact prediction			Binding site prediction							
			${\bar{AUC}}_{ROC}$	$AUC_{ROC}$	$AUC_{PR}$	${\bar{AUC}}_{ROC}$	$AUC_{ROC}$	$AUC_{PR}$	MCC	PR	RC	SPC	NPV
BIPSPI	DImS	Seq	0.7469	0.7300	0.0170	0.6883	0.6741	0.3375	0.2330	0.3592	0.4264	0.8219	0.8595
		Struc*	0.8800	0.8909	0.0432	0.7940	0.7816	0.4739	0.3679	0.4750	0.5098	0.8680	0.8832
		Struc	0.8789	0.8875	0.0439	0.7985	0.7847	0.4772	0.3779	0.4416	0.5983	0.8228	0.8974

	DBv5	Seq	0.8024	0.8137	0.0110	0.7286	0.7527	0.3049	0.2791	0.3003	0.4828	0.8349	0.9322
		Struc*	0.9011	0.9184	0.0238	0.8046	0.8154	0.3967	0.3721	0.4012	0.5079	0.9037	0.9353
		Struc	0.9052	0.9188	0.0234	0.8235	0.8225	0.4104	0.3855	0.3910	0.5585	0.8895	0.9407

	DBv3	Seq	0.8153	0.8154	0.0113	0.7361	0.7492	0.3041	0.2830	0.3233	0.4396	0.8828	0.9251
		Struc*	0.9024	0.9186	0.0269	0.8103	0.8136	0.4081	0.3712	0.4223	0.4815	0.9112	0.9287
		Struc	0.9044	0.9131	0.0234	0.8157	0.8163	0.4058	0.3730	0.3831	0.5458	0.8871	0.9383

PAIRpred	Dv3	Seq	0.809	NA	NA	0.708	0.708	NA	NA	NA	NA	NA	NA
		Struc-d	0.8783	0.8930	0.0125	0.7587	0.6913	0.2012	0.1807	0.1680	0.7809	0.5030	0.9470
		Struc-p	0.8783	0.8930	0.0125	0.7689	0.7741	0.3412	0.3112	0.3716	0.4197	0.8987	0.9256

PPiPP	Dv3	Seq	0.729	NA	NA	0.661	0.661	NA	NA	NA	NA	NA	NA

*Note*: Seq, Sequence-based features only; Struct*, Structural and sequence-based features one step; Struc, Structural and sequence-based features two steps (default). Struc-d, PAIRpred structural and sequence-based features and maximum as scoring function (default); Struc-p, PAIRpred structural and sequence-based features and proposed scoring function. NA, Not available

In general, binding site evaluation measurements improved after the second step. For example, when BIPSPI was evaluated in DBv5 the MCC in the second step increased by >0.01 respect the first step. Also, ${\bar{AUC}}_{ROC}$, $AUC_{ROC}$ and $AUC_{PR}$ measurements increased after the second step was employed (see Table 1). It is worth noting that the apparent precision drop in the second step that could be inferred from Table 1 values is a consequence of the fact that precision and recall were obtained for those thresholds that maximized the MCC in each step independently and thus, they cannot be compared. In fact, as it can be appreciated in the precision-recall curves included in Supplementary Section S6.2, most precision and recall values improved after the second step was applied. This improvement between the two steps can be explained by the addition of the first step scores and their associated structural pairwise environment scores (see Section 2.3.2). Protein binding sites tend to form continuous surface patches and thus, providing predicted scores of neighbour residues can be useful in order to find residues surrounded by high scored regions.

Furthermore, we analyzed the feature importance for the second step classifier obtaining that the first step scores and its associated environment values were the most important features (see Section 2.3.2 and Section 3.1). In addition to XGBoost algorithm, which has not been widely explored in bioinformatics, we have also analyzed Random Forest (Breiman, 2001) as classifier. Results obtained by XGBoost were superior to Random Forest in all the evaluated metrics (see Supplementary Section S7). Specifically, XGBoost achieved a higher recall (over 7%) while having a similar precision and increased the RRCP ${\bar{AUC}}_{ROC}$ over 1% and binding site MCC over 0.02.

### 3.3 BIPSPI behaves partner-specific

In order to measure the partner-specificity of BIPSPI, we have compiled a dataset where some proteins interact with multiple partners through different binding sites. Then, we have compared the scores of binding site residues for a particular interaction (e.g. protein P_A_ interacting with P_B_) with the scores of residues involved in the interaction of the same partner but with other proteins (e.g. protein P_A_ interacting with P_C_ or with P_D_). For this analysis, equivalent proteins (sequence identity >90%) that interact with different partners were identified from our datasets DBv5 and DImS. As a result, 46 different proteins, involving 123 interactions, were found in DBv5 and 17 proteins, involving 43 interactions, in DImS (see Supplementary S9.1 for a list of pdb chains). To avoid any effect or artefact due overtraining, we analyzed the scores obtained in the leave-one-out cross-validation computed on DBv5 and DImS (see Section 3.2). Then, for each protein, scores from its specific interface residues were collected for the partner-specific binding site distribution and scores from residues that belong to the interfaces of other interactions were included in the non-specific binding site distribution (see Supplementary Material Section S9.2 for a detailed explanation and a particular example). Finally, both distributions were compared using the Mann–Whitney *U* test, achieving *P*-values of 2.6e-13 and 2.5e-14 for DBv5 and DImS, respectively and thus, rejecting the null hypothesis of the test that both distributions are equivalent.

### 3.4 Binding site scoring function improves other approaches

In PPiPP and PAIRpred, the binding site score of a particular residue is computed as the mean or the maximum of the residue pair scores involving this particular residue. Then, for a single residue, the resulting binding site score depends on the score of a unique pair and thus, the predicted score of other possible contacts are ignored. In this work, we have designed a novel scoring function to compute single residue binding site scores considering all predicted score pairs for a particular residue (see Section 2.5). This approach increased the performance when compared with the maximum score value proposed in PAIRpred. Finally, we have also found that averaging the predicted binding site scores through a sliding window (see Section 2.5) increased the final performance.

Table 2 summarizes the performance of different scoring approaches predicting biding sites from residue pair scores. In our benchmark, the best performance was achieved by the newly defined scoring function averaging the resulting scores through a sliding window. At this point, we would like to highlight that our proposed scoring function is not specific for our method but also can be applied to other pair prediction methodologies. Indeed, when applied to PAIRpred scores, it also improves its performance (see Table 1 and Section 3.5).

**Table 2.** Performance evaluation for BIPSPI interface scores estimated by a leave-one-out cross-validation over the complexes compiled in DBv5 using different scoring strategies

Algorithm	Input	Binding site prediction					
		$AUC_{PR}$	MCC	PR	RC	SPC	NPV
$I_{s}(\alpha )$	Seq	0.2968	0.2740	0.3005	0.4679	0.8617	0.9272
	Struc	0.4043	0.3826	0.3947	0.5444	0.8940	0.9392
$I_{s}(\alpha )$+ wAVG	Seq	0.3049	0.2791	0.3003	0.4828	0.8349	0.9322
	Struc	0.4104	0.3855	0.3910	0.5585	0.8895	0.9407
Maximun	Seq	0.1955	0.1684	0.1761	0.6459	0.6163	0.9320
	Struc	0.3199	0.2977	0.2679	0.6394	0.7780	0.9444

*Note*: $I_{s}(\alpha )$, Proposed scoring function; $I_{s}(\alpha )$+ wAVG, Proposed scoring function followed by averaging along sequence (default); Seq, Sequence-based features only; Struct, Structural and sequence-based features two steps (default).

### 3.5 Comparison with other methods

We have compared our approach with four other methods (PPiPP, PAIRpred, GCNN and ECLAIR) that also use a machine-learning based approach and have been designed to predict partner-specific binding sites. In order to make comparisons with PAIRpred and PPiPP easier, we have used the same evaluation protocol consisting in a leave-one-out cross-validation over DBv3 complexes. Table 1 shows the performance of PPiPP, PAIRpred and BIPSPI using the metrics described in Section 2.6. The best performance was achieved by BIPSPI when structural data was included in the input data. Moreover, when only sequence-based features were used, BIPSPI also outperformed the other approaches. It is worth to highlight that original PAIRpred binding site predictions considerably improved when our scoring function was applied (see Section 2.5), raising the MCC coefficient by >0.1 points.

Comparison with GCNN was carried out as described in the original publication (Fout *et al.*, 2017). Thus, BIPSPI was retrained on the set of complexes of DBv5 that are contained in Docking Benchmark v4 (DBv4) (Hwang *et al.*, 2010) and tested on the complexes contained in DBv5 but not in DBv4. The median ROC-AUC obtained by BIPSPI on the testing set was 0.942 and thus, >4 points better than the reported in GCNN publication. Similarly, we compared our method with ECLAIR and several other non-partner-specific methods using the BM90C dataset (Meyer *et al.*, 2018). In this case, BIPSPI also achieved the best MCC when compared with the other methods, 0.389. A detailed comparison table is included in Supplementary Section S8.1.

In addition, we have also evaluated BIPSPI performance over a set of CAPRI targets (see Supplementary Section S6.12 for a complete list of proteins and detailed results). In this evaluation, BIPSPI achieved an ${\bar{AUC}}_{ROC}$ for pair prediction of 0.885 and, for binding site $AUC_{ROC}$ and MCC, values of 0.763 and 0.297, respectively. Moreover, we could compare these results with ISPRED4 predictions (Savojardo *et al.*, 2017) as these targets were also used during its testing. It is worth noting that, ISPRED4 is a non-partner-specific predictor and thus, predicting global binding sites is a more general problem. Even so, BIPSPI obtains better MCC than ISPRED4, which reported an MCC of 0.28.

### 3.6 Use case

In this section, we illustrate how BIPSPI can be employed in order to obtain meaningful information of protein–protein interfaces, especially in those cases where several partners are involved and thus, partner-specificity becomes more important. One of these examples can be found in pdb 4ov6, in which two subunits of the preprotein convertase subtilisin/kexin type 9 (PCKS9) are in complex with a PCSK9-binding adnectin protein. PCSK9 plays an important role in the regulation of low-density lipoprotein (LDL) serum levels thanks to its LDL receptor degrading activity and it has been demonstrated that self-association of PCKS9, that occurs at the catalytic region, increase that activity (Fan *et al.*, 2008). For these reasons, it has become a potential pharmacological target for the treatment and prevention of cardiovascular diseases (Mitchell *et al.*, 2014). PCSK9-binding adnectins, which were derived from human fibronectin as an alternative to therapeutic antibodies, are known to bind also close to the active site (Mitchell *et al.*, 2014).

**Fig. 2.**
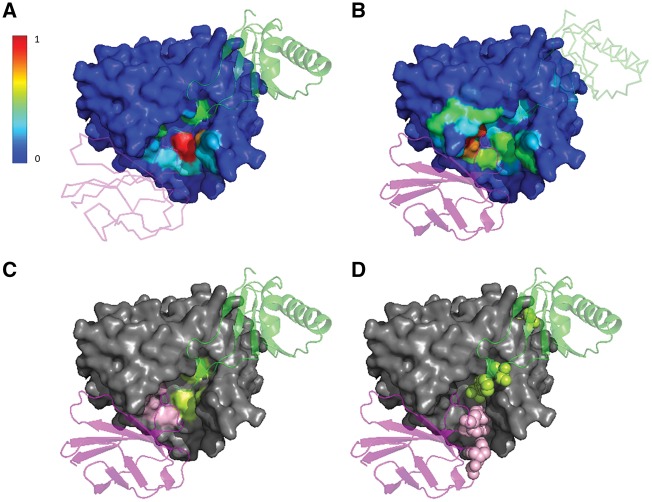
BIPSPI interface predictions for the proteins included in pdb 4ov6 bioassembly number 2. Subtilase domain of PCKS9 protein (pdb-chain E), surface representation. Peptide inhibitor domain of PCKS9 (pdb-chain D), green ribbon or trace schema. PCSK9-binding adnectin protein (pdb-chain G), magenta ribbon or trace schema. (**A**) Normalized binding site prediction scores for the prediction of the PCKS9 subtilase domain (heat map surface) interacting with the peptide inhibitor domain (green ribbon). Scores for all residues are displayed. (**B**) Normalized binding site predicted scores for the prediction of the PCKS9 subtilase domain (heat map surface) interacting with PCSK9-binding adnectin protein (magenta ribbon). Scores for all residues are displayed. (**C**) Compact representation of (A) and (B) in which just the highest score binding site residues for each interacting binding site are depicted. For the PCKS9 subtilase domain (grey surface), residues that interact with the peptide inhibitor domain (green ribbon) are coloured in lemon-green and in light-pink when they interact with the PCSK9-binding adnectin protein (magenta). (**D**) Residue spheres representation of the top four highest score residue predictions coloured in light-pink for the PCSK9-binding adnectin protein (magenta) and lemon-green for the peptide inhibitor domain (green) (Color version of this figure is available at *Bioinformatics* online.)

BIPSPI interface residue predictions for the PCKS9-PCKS9 interaction and for the PCKS9-Adnectin interaction are shown in Figure 2. As it can be observed, BIPSPI partner-specificity allows the identification of some of the residues of each native binding site, despite being spatially close. Moreover, it can be noticed that BIPSPI predictions are spatially close to the active site that was identified through 3DBIONOTES application (see Supplementary Section S10 for additional information and Section S11 for an additional use case) (Segura *et al.*, 2017; Tabas-Madrid *et al.*, 2016).

## 4 Conclusion

In this work, we have presented BIPSPI, a partner-specific predictor of residue–residue contacts and protein binding sites that uses as input either protein sequences or structures. BIPSPI employs the Extreme Gradient Boosting algorithm over a set of structural and/or sequence-based features in order to predict scores of residue pairs that are likely to interact. Then, these predicted scores are converted into binding site predictions by a novel scoring function. BIPSPI was compared with state of the art methods using a leave-one-out cross-validation on different datasets. Additionally, several CAPRI targets were also tested as an independent evaluation benchmark. In all these evaluations, BIPSPI achieved the best performance compared to previously reported methods. Moreover, its partner specificity was successfully evaluated through a Mann–Whitney *U* statistical test. Finally, BIPSPI is freely available through a user-friendly web application at http://bipspi.cnb.csic.es where prediction and visualization of binding site residues can be compute from either protein structures or sequences.

## Funding

Instituto de Salud Carlos III, project number PT13/0001/0009 and PT17/0009/0010 funding the Spanish National Institute of Bioinformatics. The Spanish Ministry of Economy and Competitiveness through Grants AIC-A-2011-0638, BIO2013-44647-R, BIO2016-76400-R(AEI/FEDER, UE), the ‘Comunidad Autónoma de Madrid’ through Grant: B2017/BMD-3817. Horizon 2020 through Grant CORBEL (INFRADEV-1-2014-1—Proposal: 654248), ELIXIR-EXCELERATE (INFRADEV-1-2015-1—Proposal: 676559) and West-Life (EINFRA-2015-1, Proposal: 675858). J. Segura is recipient of a ‘Juan de la Cierva’ fellowship and R. Sanchez-Garcia is recipient of a FPU fellowship. The authors acknowledge the support and the use of resources of Instruct, a Landmark ESFRI project.

*Conflict of Interest*: none declared.

## Supplementary Material

Supplementary Material
