# Supplementary material for: BIPSPI: a method for the prediction of partner-specific protein–protein interfaces
Source: Bioinformatics. 2018 Jul 18;35(3):470–7. doi: 10.1093/bioinformatics/bty647 (PMC6361243; doi:10.1093/bioinformatics/bty647)
Supplement: Supplementary Material [file bty647_supplementary_material.pdf]

SUPPLEMENTARY MATERIAL: TABLE OF CONTENTS

S1. Sequence-based approach 2

S1.1. Features and environments 2

S1.2. Workflow 2

S2. Detailed description of features 2

S3. Extreme Gradient Boosting Training and Hyperparameters 4

S4. Binding site scoring function 5

S4.1. Scoring function example calculation 5

S4.2. Expected precision of binding site scores 5

S5. XGBoost model features importance 6

S6. Detailed performance analysis 7

S6.1 Additional statistical measurements for all datasets 7

S6.2. ROC curves and precision-recall curves for several datasets. 8

S6.3. ROC curves and precision-recall curves for different scoring functions. 10

S6.4. Effect of negative sampling scheme in performance 11

S6.5. Effect of bound and unbound structures on training 11

S6.6. Effect of different independence criteria on training. 12

S7. XGBoost model compared to Random Forest model 12

S8. Comparison with other methods. 12

S8.1. Comparison with ECLAIR and several non-partner specific methods. 12

S8.2. Comparison with PAIRpred 13

S9. BIPSPI behaves partner-specific 14

S9.1. Groups of proteins in DBv5 and DImS sets that interact with several partners. 14

S9.2. Specific Partner Specific Scores (SPIS) and Other Partners Interface Scores (OPIS). 15

S9.3. Box plots of Specific Partner Specific Scores (SPIS) and Other Partners Interface Scores (OPIS) distributions 15

S10. Use case PCKS9-PCKS9 & PCKS9-Adnectin 16

S10.1. Predictions happen to be spatially close to protein active site. 16

S10.2. Comparison with non-partner-specific methods. 16

S11. Use case SHR-JACKDAW & SHR-SCR 17

S12. Results per complex 18

S1. Sequence-based approach

S1.1. Features and environments

Sequence binding site approach employs the same sequence-based and environment (sliding window) features that structure-based model (see Main Text Section 2.3.2). Additionally, input features include the protein sequence length in an attempt to correct the expected total number of interface residues, a similar approach was used in other works (Yuan, 2005).
When only sequence data is used, all structural features are replaced by accessibility and secondary structure predictions (three probability values) computed with SPIDER2 (Heffernan *et al.*, 2015). As no structural data is available, structural environments cannot be computed.

S1.2. Workflow

Contrary to structure-based method, which employs two XGBoost steps, sequence-based version consists of just one classifier followed by the same scoring function that the structure-based approach employs (see Main Text Section 2.4). As no second step is performed, predicted score pairs are not used as features and thus, no pairwise environment is included. Figure S1.2.1 shows a comparison of structure-based method and sequence-based method. Both approaches start by codifying pairs of amino acids using their features. After that, an XGBoost model is trained over these codified pairs. However, in the case of structure-based model, a second classifier is trained employing the predictions of the first step together with the original features. Predictions obtained by these second classifier will be considered as the final pair predictions of structure-based model. Lastly, final predicted score pairs (first step classifier for sequence-based model and second step classifier for structure-based model) are converted to binding site predictions by means of a scoring function (see Main Text Section 2.6) which is common to both workflows.

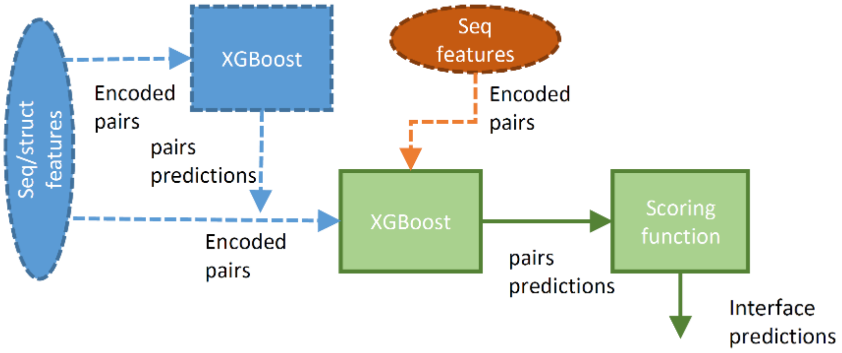

Figure S1.2.1. BIPSPI workflow. Algorithm starts at blue ellipse when structures are provided as input whereas it begins at orange ellipse when sequences are provided as input. Green part of the diagram shows the shared workflow (although XGBoost model is different as features are different).

S2. Detailed description of features

Table S2.1. Sequence-only method features per residue (total number of features is twice the number of features for one residue).

Name	# of features	Calculation procedure	Description
**One-hot encoded amino acid symbol**	22*	Custom script	Each amino acid is codified as a vector of 22 elements in which all them are zero except the element that identifies the amino acid type. Amino acid types considered are the 20 standard amino acids, non-standard amino acid and non-amino acid (for window positions

			outside the sequence).
**One-hot encoded amino acid symbol in sliding window**	242	Custom script	Window size of 11 amino acids
**PSSM profile**	20*	PSI-Blast	Each residue of the sequence is described by a vector of 20 digits
**PSFM profile**	20*	PSI-Blast	Each residue of the sequence is described by a vector of 20 digits
**Information per position**	1*	PSI-Blast	One value per residue in sequence. It is related with column entropy in multiple sequence alignment
**weight of gapless**	1*	PSI-Blast	One value per residue in sequence. It is related with column gaps in multiple sequence alignment and can indicate profiles' local quality
**MSA conservation**	1	PSI-Blast alignments and AL2CO	One value per residue in sequence. AL2CO Processes Psi-blast retrieved multiple sequence alignment to compensate for redundancy and estimates conservation score.
**Sequence profiles sliding window**	462	Custom script	Window size of 11 amino acids
**Sequence length**	1	Custom script	The sequence length of the chain where the residue belongs is included in an attempt to correct for the total expected number of interface residues. Similar approach was used in Yuan (Yuan, 2005). All residues of the same pdb chain shared the same value
**Solvent accessibility prediction**	1	SPIDER2	One value for each residue
**Secondary structure prediction**	3	SPIDER2	Three probability values (0-1 range) for each residue

Notes: * not used directly for pair codification as they are included in sliding window

Table S2.2. Structure-based method features per residue (total number of features is twice the number of features for one residue).

Name	# of features	Calculation procedure	Description
**One-hot encoded amino acid symbol**	22	Custom script	Each amino acid is codified as a vector of 22 elements in which all them are zero except the element that identifies the amino acid type. Amino acid types considered are the 20 standard amino acids, non-standard amino acid and non-amino acid (for window positions outside the sequence).
**One-hot encoded amino acid symbol in sliding window**	242	Custom script	Window size of 11 amino acids
**One-hot encoded amino acid symbol in structural environment**	22	Custom script	
**PSSM profile**	20	PSI-Blast	Each residue of the sequence is described by a vector of 20 digits
**PSFM profile**	20	PSI-Blast	Each residue of the sequence is described by a vector of 20 digits
**Information per position**	1	PSI-Blast	One value per residue in sequence. It is related with column entropy in multiple sequence alignment
**weight of gapless**	1	PSI-Blast	One value per residue in sequence. It is related with column gaps in multiple sequence alignment and can indicate profiles' local quality
**Conservation**	1	PSI-Blast alignments and AL2CO	One value per residue in sequence. AL2CO Processes Psi-blast retrieved multiple sequence alignment to compensate for redundancy and estimates conservation score.
**Sequence conservation in sliding window**	462	Custom script	Window size of 11 amino acids
**Sequence conservation in structural**	172	Custom script	

**environment**			
**Solvent accessibility**	10	PSAIA	
**Hydrophobicity**	1	PSAIA	
**Depth index**	6	PSAIA	
**Protrusion index**	6	PSAIA	
**PSAIA in structural environment**	92	Custom script	
**One-hot encoded secondary structure**	8	DSSP	Each residue is codified as a vector of 8 elements in which all them are zero except the element that identifies the secondary structure type (7 defined secondary structure types by DSSP and 1 for no detected secondary structure).
**One-hot encoded secondary structure in structural environment**	8	Custom script	Window size of 11 amino acids
**Half-sphere exposure**	2	Biopython	Each residue is codified by 2 numeric values that represent half-sphere exposure computed by approximate CA-CB vectors and by exact CA-CB vectors
**Contact number**	1	Biopython	Number of CA around a residue with max distance= 12 Å
**Half-sphere exposure and contact number in structural environment**	12	Custom script	

Table S2.3. Structure-based method pairwise features per residue-residue pair in second step classifier

Name	# of features	Calculation procedure	Description
**Previous step predictions**	2	Custom script	Each pair of amino acids is codified employing the prediction obtained by first classifier (1 value) and the same prediction normalized (mean, standard deviation) over all pairs contained in the complex.
**Previous step predictions in structural pairwise environment**	24	Custom script	From both raw first step prediction and normalized prediction, 4 new values (maximum, minimum, sum and mean) for each type of environment ($\alpha\ vs\ N_\beta$, $N_\alpha\ vs$ β and $N_\alpha\ vs\ N_\beta$).

S3. Extreme Gradient Boosting Training and Hyperparameters

We have trained one XGBoost model per protein complex for each of the steps of the algorithm. Thus, each model was trained on the concatenation of all codified pairs but the ones that belong to the left-out complex. Predictions for all pairs in a complex obtained in first step leave-one-out were used as features for the second step.

XGBoost hyperparameters of Python2.7 xgboost package on sklearn API were set to default with the exception of the following, which were set to:

- 'objective':'binary:logistic'
- 'colsample_bytree': 0.9,
- 'learning_rate': 0.1
- 'min_child_weight': 1
- 'n_estimators': 2000
- 'subsample': 0.9
- 'reg_lambda': 10.0
- 'max_depth': 12
- 'gamma': 0

S4. Binding site scoring function

S4.1. Scoring function example calculation

Table S4.1. Hypothetical predictions for residue pair interaction results obtained by XGBoost model. Rows are sorted from highest to lowest scores.

ResId_protA	ResId_protB	Pairs score
Ala32	Asn1	0.901
Asp15	Ile6	0.887
Val2	Met11	0.805
Ala32	His12	0.799
Ala32	Ile6	0.779
Phe1	Ser4	0.751
Ala32	Ser4	0.779
Phe1	Asn1	0.751
Ala34	Thr46	0.779
Phe1	Asn67	0.751
…	…	…

In this section, 5 different examples of interface score computation will be shown using as interacting pairs scores the ones shown in Table S4.1. In order to obtain interface scores, table should be sorted from highest to lowest by Pairs scores (which is the case). Then, interface scores are computed independently for each protein by counting the number of times a given residue appears among the top k highest score pairs for k=2^1,2^2… and dividing this count over k.

$$I_S(\text{"protA Ala32"}) = X_C\left(\text{protA Ala32}, 2^1\right) + X_C\left(\text{protA Ala32}, 2^2\right) + X_C\left(\text{protA Ala32}, 2^3\right) + \cdots = \frac{1}{2} + \frac{2}{4} + \frac{4}{8} + \cdots$$
$$I_S(\text{"protA Asp15"}) = X_C\left(\text{protA Asp15}, 2^1\right) + X_C\left(\text{protA Asp15}, 2^2\right) + X_C\left(\text{protA Asp15}, 2^3\right) + \cdots = \frac{1}{2} + \frac{1}{4} + \frac{1}{8} + \cdots$$
$$I_S(\text{"protA Phe1"}) = X_C\left(\text{protA Phe1}, 2^1\right) + X_C\left(\text{protA Phe1}, 2^2\right) + X_C\left(\text{protA Phe1}, 2^3\right) + \cdots = \frac{0}{2} + \frac{0}{4} + \frac{2}{8} + \cdots$$
$$I_S(\text{"protB Asn1"}) = X_C\left(\text{protA Asn1}, 2^1\right) + X_C\left(\text{protA Asn1}, 2^2\right) + X_C\left(\text{protA Asn1}, 2^3\right) + \cdots = \frac{1}{2} + \frac{1}{4} + \frac{2}{8} + \cdots$$
$$I_S(\text{"protB Ser4"}) = X_C\left(\text{protA Ser4}, 2^1\right) + X_C\left(\text{protA Ser4}, 2^2\right) + X_C\left(\text{protA Ser4}, 2^3\right) + \cdots = \frac{0}{2} + \frac{0}{4} + \frac{2}{8} + \cdots$$

S4.2. Expected precision of binding site scores

Binding site raw scores computed by BIPSPI are difficult to interpret. Although residues that exhibit high scores are more likely to belong to the interface that low score residues, it is difficult to decide which threshold might be appropriate to select the residues that comprise the predicted binding site. Consequently, we have associated to each score an expected precision value that was estimated from the measured scores vs. precision data resulted from BIPSPI benchmarking (see Figure S4.2.1). In order to model the relation between score and precision, two well-known techniques were tried: Platt's sigmoid fit (Platt and Platt, 1999) and isotonic regression (Zadrozny and Elkan, 2002). As it can be appreciated in Figure S4.2.1, sigmoid fit was overoptimistic for most score values and it was also not able to explain well the range of low score values. On the contrary, isotonic regression model was better fitted to data, and thus, this latter approach was employed in BIPSPI web server.

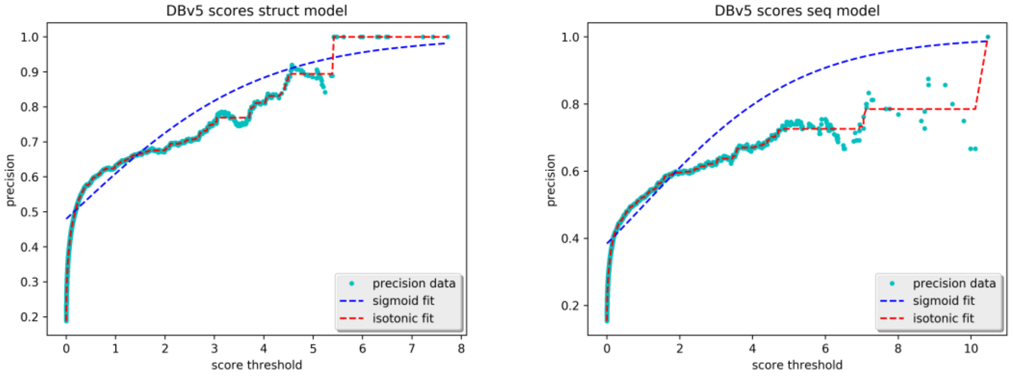

Figure S4.2.1. Binding site scores of residues against measured precision (red) and isotonic fit to data (blue). Scores for structure-based model are displayed in the left hand side plot, sequence-based model in the right hand side.

In Figure S4.2.2, the distribution of binding site precision per complex is displayed for several expected precision thresholds (raw score transformed into expected precision by isotonic regression). As it can be appreciated, most complexes exhibit precision levels close to the expected precision threshold.

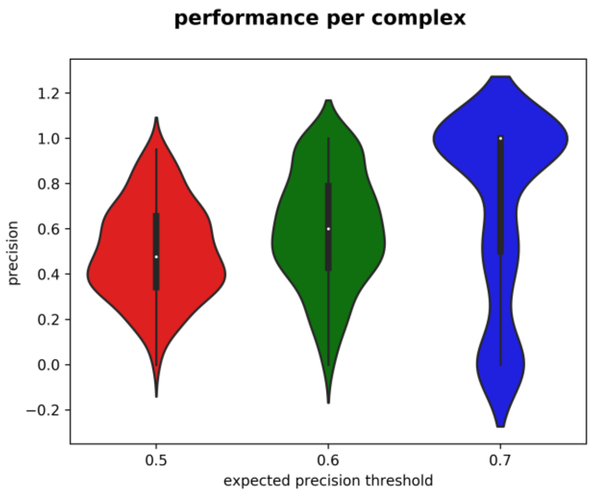

Figure S4.2.2. Precision per DBv5 complex for three distinct score thresholds associated with expected precisions of 0.5, 0.6 and 0.7 respectively.

S5. XGBoost model features importance

Different approaches can be used to measure feature importance in tree ensemble methods. Some of them employ the average information gain associated to a feature when it is used in each tree, some others take into account mean impurity decrease for each tree split and feature, etc. One common strategy, which was used in this work, is to sum the number of times each feature is responsible for a tree split. Thus, feature importance for each variable was determined as the number of times the variable was used in a tree split divided by the total number of splits that occurred in all trees. As a result, features importance was expressed as a relative value ranged from 0-1 such that the importance of all variables sums up to 1.

As our model makes use of around 2000 input variables, many of them representing the same concept (e.g., 506 one-hot encoded variables describe amino acid type, including sliding window and structural environment), feature importance was determined for groups of related features (e.g. Accessibility, Conservation, etc.). In each group, both single amino acid and environment features (see Main Text Section 2.3.2) are included.

We defined the global feature importance of a group as the sum of relative importance of each variable that belongs to the group. Also, we defined the mean feature importance of a group as the mean of relative importance of each variable that belongs to the group. This distinction is fundamental as some groups of variables have hundreds of members (for instance sliding window of PSSM profiles) whereas some others have a few ones (for example hydrophobicity) and thus, highly populated groups tend to have higher global importance even though particular variables of the group may not be informative.

Results of feature importance analysis are shown in Figure S5.1 As it can be appreciated, globally, the most informative feature in both first step and second step classifiers is conservation information, which groups together PSSM, PSFM, Information per position, weight of gapless and AL2CO conservation score. However, this group is not so important when mean importance is considered instead. The reason for this behaviour is that more than half of the total number of variables belongs to this group. Still, the mean importance of this group is over 50% the importance of the highest important group for first step classifier, which indicates that conservation variables are informative even if less variables (smaller sliding window for instance) are included.

In terms of mean importance, the most important feature group in second step classifier is the prediction scores obtained from the first step classifier. This group of features, which involves 24 variables of the model, not only is twice as informative as the second most informative variable in terms of mean importance, but also contributes with more than 6% of the total information of the model (around 1% will be

expected if all variables contributed equally). These results may explain why second step classifier works better than first step classifier.

Apart from first step predictions, the group of variables with better mean importance is accessibility, which also explains an important amount of global importance. This is not shocking as in Minhas et al. (Minhas *et al.*, 2014) it was demonstrated that solvent accessibility alone has the capability to predict interacting residue pairs with certain reliability.

Additionally, we have studied the importance of the different types of environment variables, independently of its nature. As it can be appreciated in Figure S5.1, the global contribution of structural environment variables is greater than 55% of total importance even though this group represents 31% of the total number of variables. Consequentially, the mean importance of structural environment features is about three times bigger than the mean importance of sliding window environment, which confirms that the structural environment of a pair of amino acids contributes massively to the prediction.

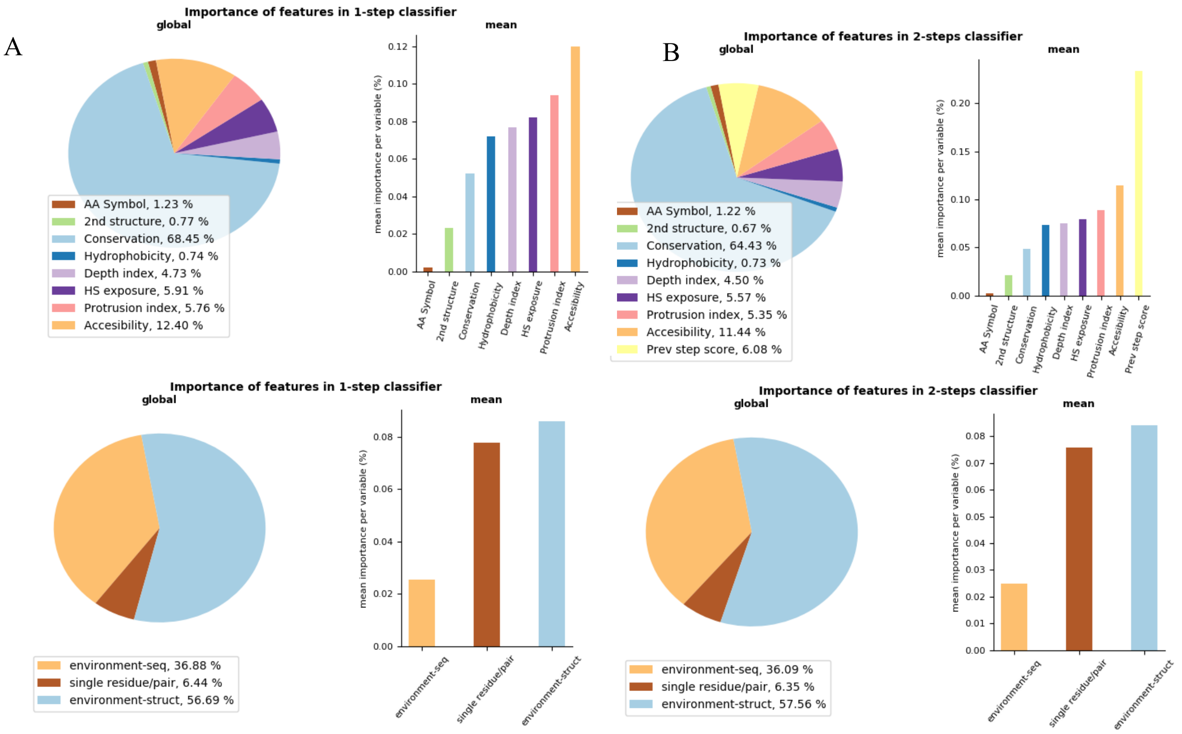

Figure S5.1. Importance of features employed in BIPSPI for first step classifier (A) and second step classifier (B). Pie charts show the relative importance of groups of variables as percentage of total importance, whereas bar plots display the relative importance of each group of features as a percentage.

S6. Detailed performance analysis

S6.1 Additional statistical measurements for all datasets

Table S6.1.1. Performance summary of BIPSPI on several datasets.

Data	Algorithm	Input type	Residue-Residue Contact Prediction				Binding Site Prediction								
			$\overline{\text{AUC}}_{ROC}$	AUC_{ROC}	$\overline{\text{AUC}}_{PR}$	AUC_{PR}	$\overline{\text{AUC}}_{ROC}$	AUC_{ROC}	$\overline{\text{AUC}}_{PR}$	AUC_{PR}	MCC	PR	RC	SPC	NPV
DBv5	B	Seq	0.8024	0.8137	0.0371	0.0110	0.7286	0.7527	0.3539	0.3049	0.2791	0.3003	0.4828	0.8349	0.9322
	B-1	Struc	0.9011	0.9184	0.0586	0.0238	0.8046	0.8154	0.4438	0.3967	0.3721	0.4012	0.5079	0.9037	0.9353
	B	Struc	0.9052	0.9188	0.0642	0.0234	0.8235	0.8225	0.4629	0.4104	0.3855	0.3910	0.5585	0.8895	0.9407
	B max	Seq	0.8024	0.8137	0.0371	0.0110	0.7234	0.6809	0.3219	0.1955	0.1684	0.1761	0.6459	0.6163	0.9320
	B max	Struc	0.8024	0.8137	0.0371	0.0110	0.8177	0.7947	0.4394	0.3199	0.2977	0.2679	0.6394	0.7780	0.9444

	B -wAvg	Seq	0.8024	0.8137	0.0371	0.0110	0.7205	0.7386	0.3462	0.2968	0.2740	0.3005	0.4679	0.8617	0.9272
	B -wAvg	Struc	0.9011	0.9184	0.0586	0.0238	0.8043	0.8092	0.4568	0.4043	0.3826	0.3947	0.5444	0.8940	0.9392
DBv3	B	Seq	0.8153	0.8154	0.0381	0.0113	0.7361	0.7492	0.3555	0.3041	0.2830	0.3233	0.4396	0.8828	0.9251
	B	Struc	0.9044	0.9131	0.0715	0.0234	0.8157	0.8163	0.4634	0.4058	0.3730	0.3831	0.5458	0.8871	0.9383
	PR	Seq	0.809	NA	NA	NA	NA	0.708	NA	NA	NA	NA	NA	NA	NA
	PR	Struc	0.8783	0.8930	0.0370	0.0125	0.7587	0.6913	0.3665	0.2012	0.1807	0.1680	0.7809	0.5030	0.9470
	PR-sc	Struc	0.8783	0.8930	0.0370	0.0125	0.7689	0.7741	0.3936	0.3412	0.3112	0.3716	0.4197	0.8987	0.9256
	PP	Seq	0.729	NA	NA	NA	NA	0.661	NA	NA	NA	NA	NA	NA	NA
DImS	B	Seq	0.7469	0.7300	0.0449	0.0170	0.6883	0.6741	0.3970	0.3375	0.2330	0.3592	0.4264	0.8219	0.8595
	B-1	Struc	0.8800	0.8909	0.0839	0.0432	0.7940	0.7816	0.5312	0.4739	0.3679	0.4750	0.5098	0.8680	0.8832
	B	Struc	0.8789	0.8875	0.0899	0.0439	0.7985	0.7847	0.5404	0.4772	0.3779	0.4416	0.5983	0.8228	0.8974

Note: $\overline{\text{AUC}}_{ROC}$: ROC-AUC averaged over all complexes; AUC_{ROC}: ROC-AUC for all scored pooled across complexes; $\overline{\text{AUC}}_{PR}$: Precision-recall-AUC averaged over all complexes; AUC_{PR}: Precision-recall-AUC for all scored pooled across complexes; MCC: Matthews correlation coefficient; PR: Precision; RC recall; SPC specificity; NPV negative predictive value are computed at the threshold that maximizes the Matthews correlation coefficient (MCC). Algorithms: B: BIPSPI default, B-1: BIPSPI just one step; B-max: BIPSPI 2 steps and maximum as scoring function, B – wAvg: BIPSPI 2 steps proposed scoring function but no sequence average; PR: PAIRpred; PR-sc: PAIRpred using the proposed scoring function; PP: PPiPP.

S6.2. ROC curves and precision-recall curves for several datasets.

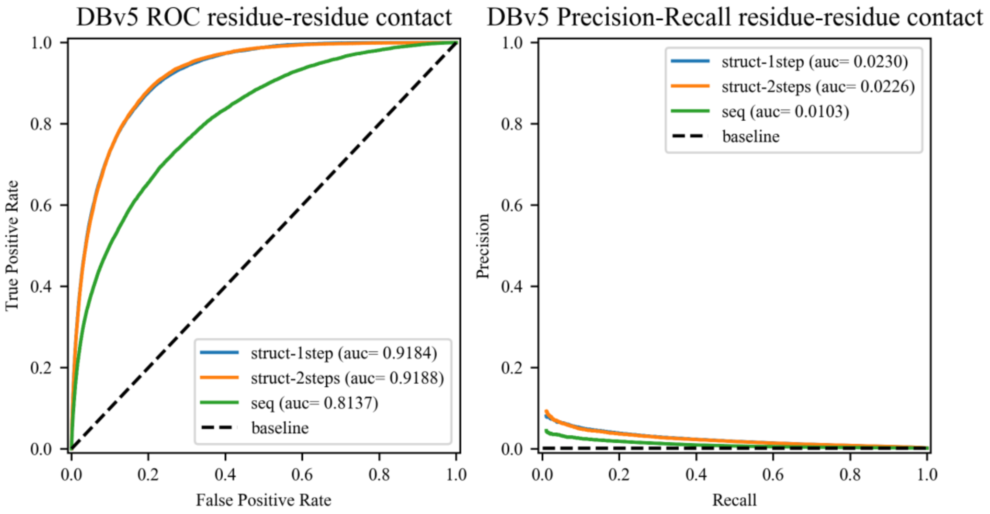

Figure S6.2.1. ROC and precision-recall curves for residue-residue predictions (all scores mixed) in DBv5. Sequence-only model is displayed in green. One-step model is displayed in blue. Two-steps model is shown in orange. Area under the curve is shown in parenthesis.

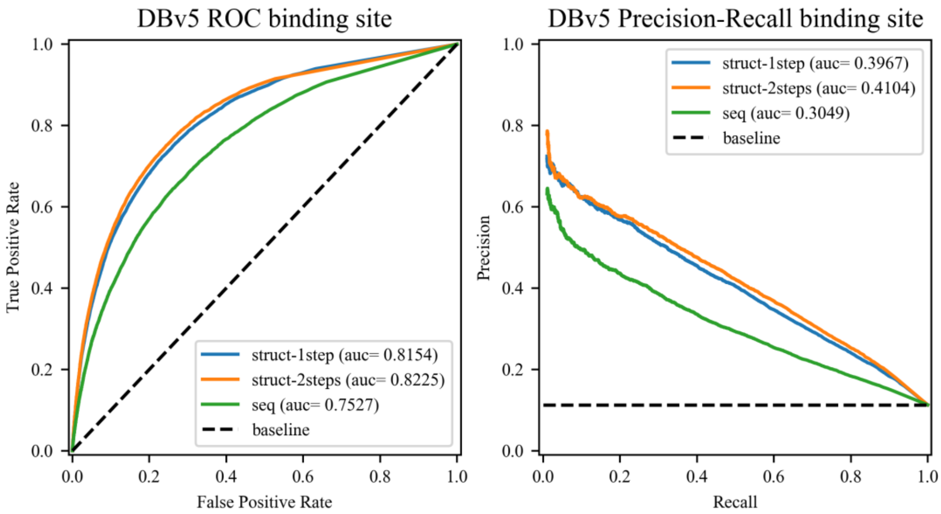

Figure S6.2.2. ROC and precision-recall curves for binding site predictions (all scores mixed) in DBv5. Sequence-only model is displayed in green. One-step model is displayed in blue. Two-steps model is shown in orange. Area under the curve is shown in parenthesis.

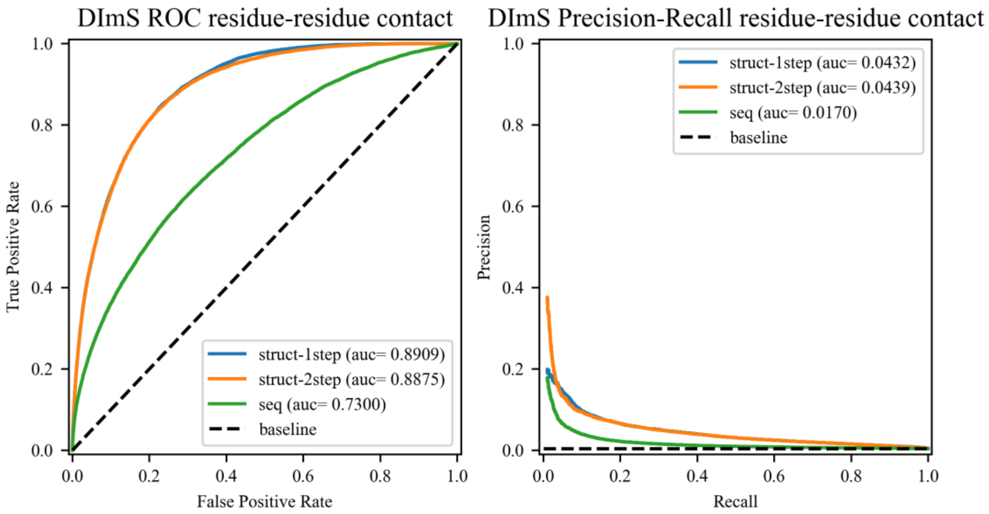

Figure S6.2.3. ROC and precision-recall curves for residue-residue predictions (all scores mixed) in DImS. Sequence-only model is displayed in green. One-step model is displayed in blue. Two-steps model is shown in orange. Area under the curve is shown in parenthesis.

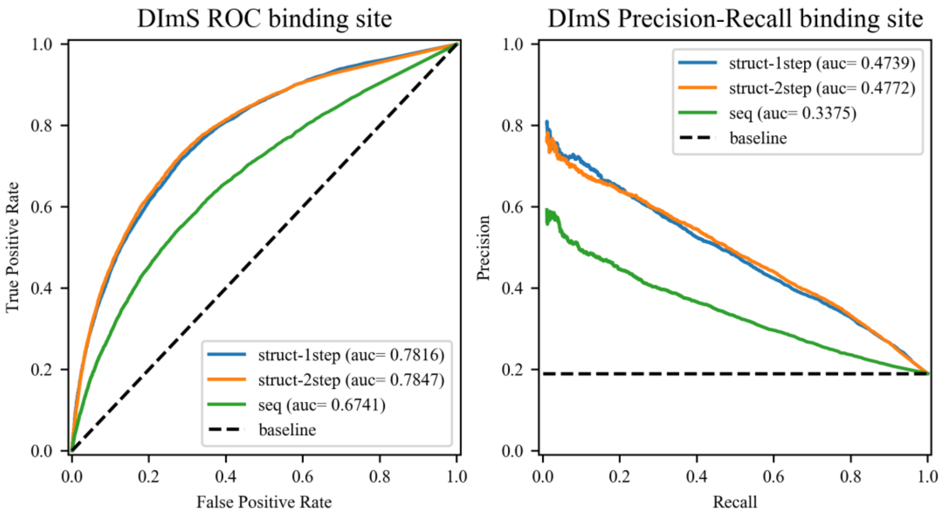

Figure S6.2.4. ROC and precision-recall curves for binding site predictions (all scores mixed) in DImS. Sequence-only model is displayed in green. One-step model is displayed in blue. Two-steps model is shown in orange. Area under the curve is shown in parenthesis.

S6.3. ROC curves and precision-recall curves for different scoring functions.

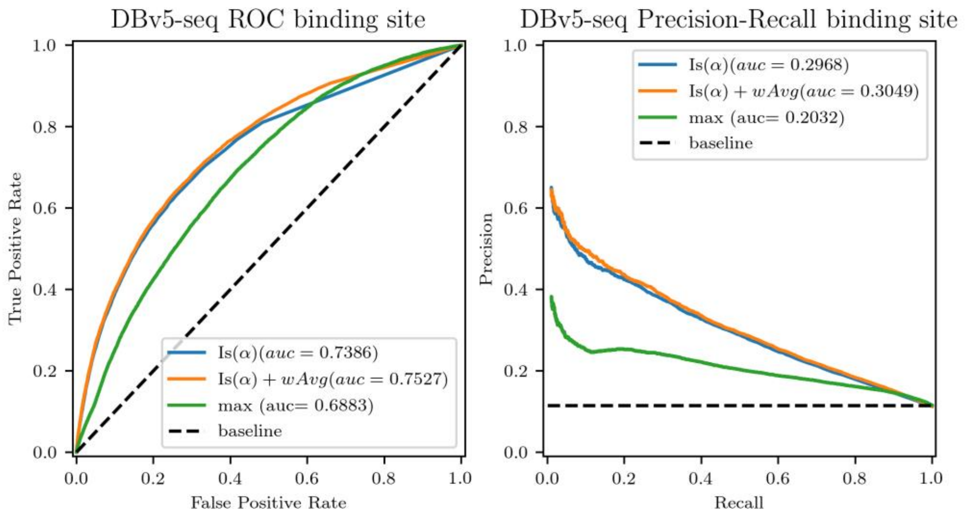

Figure S6.3.1. ROC and precision-recall curves for binding site predictions (all scores mixed) in DBv5 sequence only model. Default approach (our scoring function and sequence window average) is shown in orange. Scoring function and not window average is shown in blue. Maximum as scoring function is shown in green. Area under the curve is shown in parenthesis.

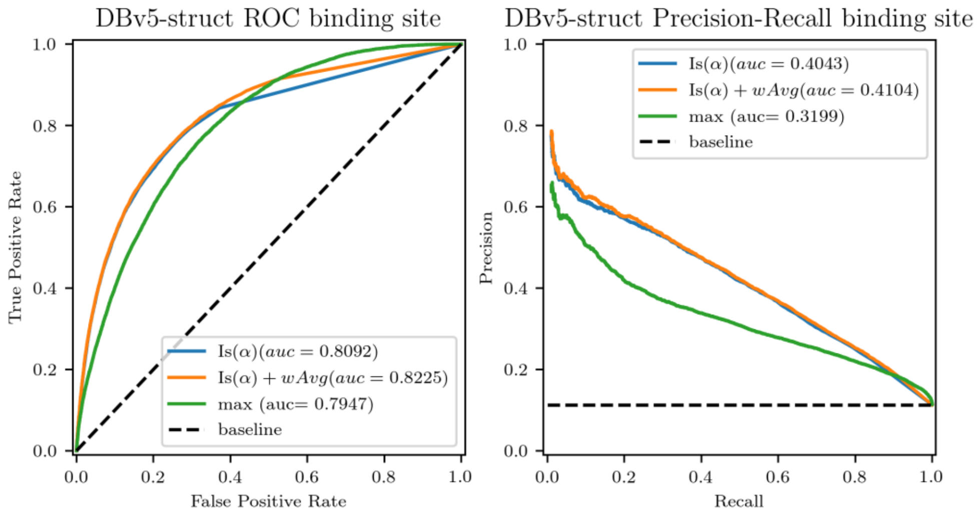

Figure S6.3.2. ROC and precision-recall curves for binding site predictions (all scores mixed) in DBv5 structure-based model. Default approach (our scoring function and sequence window average) is shown in orange. Scoring function and not window average is shown in blue. Maximum as scoring function is shown in green. Area under the curve is shown in parenthesis.

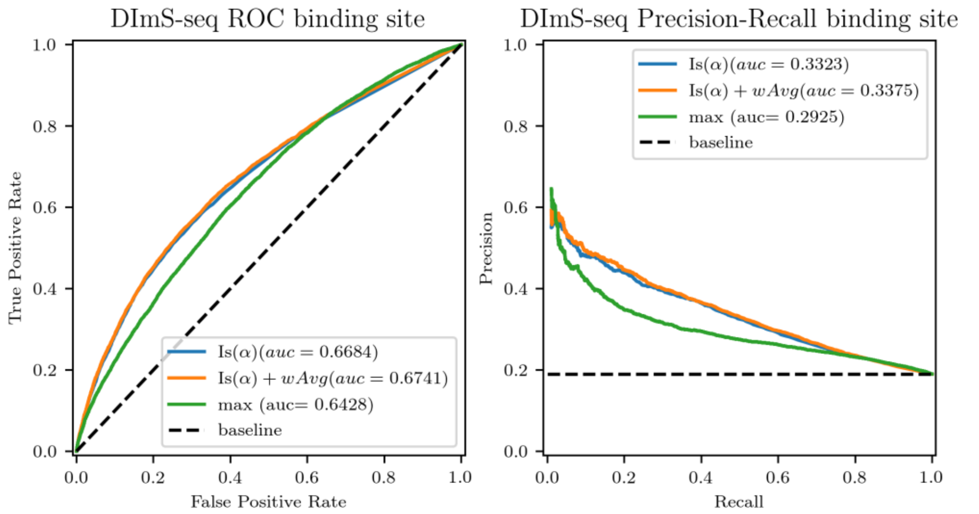

Figure S6.3.3. ROC and precision-recall curves for binding site predictions (all scores mixed) in DImS sequence only model. Default approach (our scoring function and sequence window average) is shown in orange. Scoring function and not window average is shown in blue. Maximum as scoring function is shown in green. Area under the curve is shown in parenthesis.

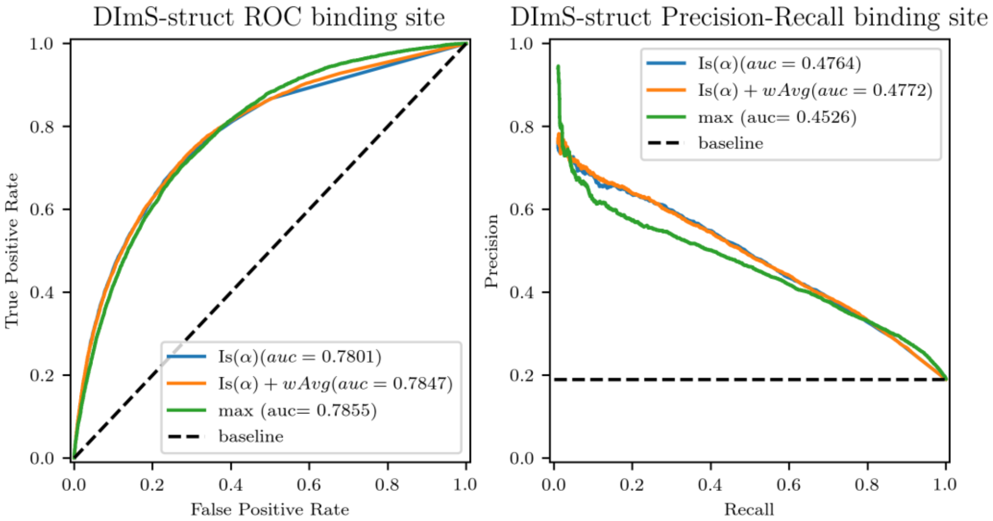

Figure S6.3.4. ROC and precision-recall curves for binding site predictions (all scores mixed) in DImS structure-based model. Default approach (our scoring function and sequence window average) is shown in orange. Scoring function and not window average is shown in blue. Maximum as scoring function is shown in green. Area under the curve is shown in parenthesis.

S6.4. Effect of negative sampling scheme in performance

For training, our method employs negative pairs sampled independently of their accessibility because otherwise, the classifier might be unable to deal with conformational changes (even small ones). For testing we use all possible pairs. We have also tried an alternative sampling criterion in which just accessible negative pairs are considered, obtaining slightly worse results.

Table S6.4.1. BIPSPI performance on DBv5 when using as negative pairs a random sample of all negative pairs and a random sample of all accessible negative pairs.

Data	Negative pairs sampling	Input type	Residue-Residue Contact Prediction				Binding Site Prediction							
			$\overline{AUC}_{ROC}$	AUC_{ROC}	$\overline{AUC}_{PR}$	AUC_{PR}	$\overline{AUC}_{ROC}$	AUC_{ROC}	AUC_{PR}	MCC	PR	RC	SPC	NPV
DBv5	Sample all	Struc	0.9052	0.9188	0.0642	0.0234	0.8235	0.8225	0.4104	0.3855	0.3910	0.5585	0.8895	0.9407
	Sample accessible	Struc	0.8993	0.9133	0.0657	0.0239	0.8062	0.8172	0.4044	0.3792	0.4054	0.5171	0.9037	0.9365

S6.5. Effect of bound and unbound structures on training

Original DBv5 results were computed using unbound structures for both training and testing. On the contrary, DImS results were obtained using bound structures for both training and testing. We have repeated this last procedure for DBv5, observing, as expected, better performance, probably because conformational changes are not present. Additionally, we have performed a cross-validation using DBv5 bound structures for training and unbound structures for testing. Interestingly, the performance under these circumstances is comparable to the performance when trained on unbound and tested on unbound structures. This suggests that bound data can be used to train our algorithm with almost the same affectivity than unbound data (which may allow for bigger training sets).

Table S6.5.1. BIPSPI performance on DBv5 when trained on bound or unbound structures and tested in unbound or bound structures.

Data	Train/Test state	Input type	Residue-Residue Contact Prediction				Binding Site Prediction							
			$\overline{AUC}_{ROC}$	AUC_{ROC}	$\overline{AUC}_{PR}$	AUC_{PR}	$\overline{AUC}_{ROC}$	AUC_{ROC}	AUC_{PR}	MCC	PR	RC	SPC	NPV
DBv5	U/U default	Struc	0.9052	0.9188	0.0642	0.0234	0.8235	0.8225	0.4104	0.3855	0.3910	0.5585	0.8895	0.9407

	B/B	Struc	0.9125	0.9278	0.0721	0.0276	0.8277	0.8278	0.4296	0.4004	0.3944	0.5999	0.8791	0.9415
	B/U	Struc	0.9030	0.9192	0.0653	0.0249	0.8229	0.8217	0.4073	0.3855	0.3713	0.6002	0.8737	0.9442

NOTE: U: unbound; B: Bound

S6.6. Effect of different independence criteria on training.

By default, BIPSPI was trained and evaluated using a leave one out procedure over DBv5. However, there is some redundancy in this dataset and thus, we have also studied how this redundancy can produce an overoptimistic view of the performance. To do so, we have performed a leave-one-group-out cross-validation in two different modes:

1) complexes which share a pair of SCOP families are left out together

2) complexes that share any SCOP family are left out together.

Results are displayed in table S.6.7.2. As expected, the default leave-one-out obtains the best performance. However, it is also worth noting that even under the strict conditions of case 2, BIPSPI performance is still close to the original benchmark, which suggest that the original results are not an artefact caused by the Docking Benchmark datasets.

Table S6.6.1. BIPSPI performance on DBv5 when trained using different independency criteria.

Data	Out-strategy	Input type	Residue-Residue Contact Prediction				Binding Site Prediction							
			$\overline{\mathrm{AUC}}_{\mathrm{ROC}}$	$\mathrm{AUC}_{\mathrm{ROC}}$	$\overline{\mathrm{AUC}}_{\mathrm{PR}}$	$\mathrm{AUC}_{\mathrm{PR}}$	$\overline{\mathrm{AUC}}_{\mathrm{ROC}}$	$\mathrm{AUC}_{\mathrm{ROC}}$	$\mathrm{AUC}_{\mathrm{PR}}$	MCC	PR	RC	SPC	NPV
DBv5	default	Struc	0.9052	0.9188	0.0642	0.0234	0.8235	0.8225	0.4104	0.3855	0.3910	0.5585	0.8895	0.9407
	SCOP pairs	Struc	0.8991	0.9169	0.0583	0.0236	0.8012	0.8139	0.3959	0.3856	0.3927	0.5564	0.8902	0.9402
	SCOP monomer	Struc	0.8933	0.8982	0.0480	0.0168	0.7956	0.8026	0.3660	0.3554	0.3643	0.5402	0.8789	0.9370

S7. XGBoost model compared to Random Forest model

Table S7.1. Performance evaluation for BIPSPI leave-one-out over the sequences and structures compiled in DBv5. XGB stands for Extreme Gradient Boosting Classifier (XGBoost) whereas RF stands for Random Forest.

Dataset	Input type	Classifier	Pairs prediction	Interface prediction			
			Mean ROC AUC	ROC AUC	MCC	Precision	Recall
DBv5	Sequence	XGB	0.8022	0.7522	0.2791	0.3003	0.4828
		RF	0.7928	0.7368	0.2626	0.2845	0.4778
	Structure	XGB	0.9011	0.8154	0.3721	0.4012	0.5079
	Structure (2-steps)	XGB	0.9052	0.8225	0.3855	0.3910	0.5585
		RF	0.8941	0.8092	0.36456	0.3980	0.4957

S8. Comparison with other methods.

S8.1. Comparison with ECLAIR and several non-partner specific methods.

Table S8.1.1 Binding site prediction performance of several methods evaluated on benchmark BM90C. Statistics are measured at the threshold that maximizes MCC.

Web server	MCC	TPR	FPR	SPC	PPV	ACC
BIPSPI	0.389	0.589	0.144	0.856	0.418	0.816
PredUs	0.383	0.701	0.156	0.843	0.302	0.831
eFindSitePPI	0.375	0.396	0.045	0.954	0.459	0.905
ECLAIR	0.322	0.346	0.041	0.959	0.431	0.909
con-PPISP	0.247	0.279	0.052	0.947	0.338	0.888
SPPIDER	0.173	0.340	0.125	0.875	0.208	0.827
ProMate	0.165	0.526	0.295	0.704	0.210	0.684
WHISCY	0.164	0.130	0.025	0.975	0.334	0.900
PIER	0.118	0.066	0.012	0.987	0.342	0.906
VORFFIP	0.117	0.531	0.401	0.598	0.337	0.579
PSIVER	0.103	0.645	0.463	0.536	0.118	0.546
InterProSurf	0.100	0.435	0.291	0.709	0.163	0.677

S8.2. Comparison with PAIRpred

Table S8.2.1 Rank of the first positive pair (RFPP) for BIPSPI and PAIRpred.

Dataset/RFPP	method	10%	25%	50%	75%	90%
DBv3	Seq PAIRpred	2	13	68	257	804
	Seq BIPSPI	2	9	41	161	817
	Struct PAIRpred	1	3	16	103	272
	Struct BIPSPI-1	1	2	11	59	238
	Struct BIPSPI	1	3	20	117	708
DBv4	Seq PAIRpred	NA	NA	NA	NA	NA
	Seq BIPSPI	2	9	41	161	817
	Struct PAIRpred	1	3	18	101	282
	Struct BIPSPI-1	2	2	13	63	219
	Struct BIPSPI	1	2	15	100	487

Table S8.2.2. Precision and recall considering as true contacts the top score pairs for BIPSPI and PAIRpred.

		Top 1%		Top 5%		Top 10%	
Dataset	method	precision	recall	precision	recall	precision	recall
DBv3	Struct PAIRpred	0.042335	0.165768	0.025129	0.464619	0.017435	0.627802
	Struct BIPSPI-1	0.067526	0.227849	0.032669	0.546392	0.021015	0.697806
	Struct BIPSPI	0.071557	0.237622	0.032260	0.560760	0.020264	0.712324
DBv4	Struct PAIRpred	0.045031	0.163094	0.032434	0.458239	0.024240	0.623560
	Struct BIPSPI-1	0.070970	0.224345	0.033533	0.535228	0.021586	0.688209
	Struct BIPSPI	0.073836	0.231141	0.034843	0.548833	0.022096	0.702792

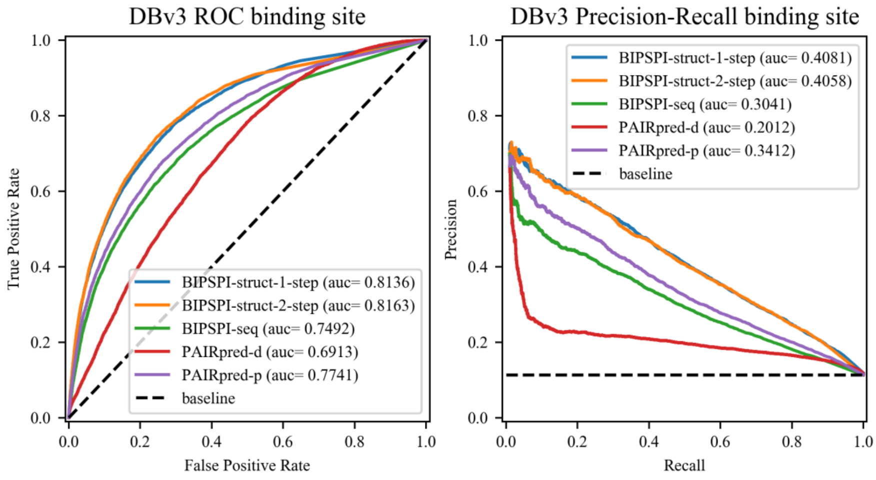

Figure S8.2.1. ROC and precision-recall curves for binding site predictions (all scores mixed) in DBv3. BIPSPI Sequence-only model is displayed in green. BIPSPI structure model 1 step is displayed in blue and 2 steps in orange. PAIRpred-d, green, are PAIRpred original scores (max). PAIRpred-p, red are PAIRpred scores computed with our scoring function. Area under the curve is shown in parenthesis.

S9. BIPSPI behaves partner-specific

S9.1. Groups of proteins in DBv5 and DImS sets that interact with several partners.

DBv5 groups of equivalent proteins that interact with different partners:
1: 1BVN_* 1KXQ_*
2: 1TMQ_* 1CLV_A
3: 3DAW_A 4H03_A 2BTF_B 1ATN_B 1H1V_B 1Y64_A 1KXP_B
4: 1GP2_* 2GTP_A
5: 4CPA_A 2ABZ_A
6: 2C0L_A 3R9A_A
7: 4M76_A 1GHQ_* 3D5S_A
8: 1FQ1_A 1BUH_*
9: 2SIC_* 2SNI_A
10: 1R0R_E BOYV_A 1OYV_A
11: 2I9B_A 2FD6_A
12: 3AAD_A BAAD_A
13: 1JTD_A 1JTG_A
14: 1GL1_1 1ACB_B 1CGI_B
15: 2OUL_A 1YVB_A
16: 1PPE_* 1OPH_A 1D6R_* 2UUY_A
17: 3EO1_A 1IQD_A
18: 3HMX_L 3EOA_L 1JPS_L 4G6M_L 1BJ1_L 3HI6_L
19: 1DQJ_C 1MLC_A
20: 4FQI_L 4GXU_L
21: 2W9E_L 2FD6_L 1E6J_L
22: 1FC2_A 1FCC_A 1FCC_B 1E4K_A 1E4K_B
23: 1M10_* 1IJK_*
24: 1IBR_A 1A2K_A 1I2M_A 1K5D_A
25: 1FAK_B 1JPS_B 1AHW_A
26: 2H7V_A 1HE1_* 2FJU_A 1I4D_* 1E96_* 2NZ8_A
27: 1J2J_A 1R8S_A
28: 3HI6_A 1MQ8_A 3EOA_A
29: 1LFD_A 1HE8_* 1BKD_A 1WQ1_D
30: 3H2V_A 1RKE_A
31: 3AAD_A BAAD_A
32: 4G6J_A 4G6M_A
33: 1MLC_* 1VFB_* 2I25_A 1DQJ_* 1BVK_*
34: 1JZD_A 1Z5Y_A
35: BOYV_A 1OYV_A
36: 3G6D_A 3L5W_A
37: 1QFW_B 9QFW_B
38: CP57_A BP57_A 3P57_A
39: 1F6M_A 2O8V_A
40: 1AKJ_E 1DE4_B
41: 3P57_B CP57_B CP57_A 3P57_A BP57_A BP57_B
42: 1QFW_A 9QFW_A
43: 2OOB_A 1S1Q_A 2AYO_A 1XD3_A
44: 4DN4_A 1ML0_*
45: 1M27_A 1EFN_A
46: 3SGQ_A 1R0R_I

DImS groups of equivalent proteins that interact with different partners:

1: 1luj_A 1jdh_A
2: 1r0r_E 1cse_E

3: 1ta3_A 1te1_A
4: 1gl1_B 1acb_E
5: 2fi4_E 1f2s_E 1tgs_Z 2uuy_A 2tld_E 1oph_B
6: 1nf3_A 1gzs_A
7: 4sgb_E 1sgp_E
8: 1cxz_A 1tx4_B
9: 1e96_A 1he1_C 1ds6_A
10: 1zc3_A 1uad_A
11: 2uyz_A 2grn_A
12: 1uuz_D 1sq2_L
13: 1b6c_C 3fap_A
14: 1xd3_B 2j7q_D 1wrd_B 1s1q_B 2g45_B 1nbf_D
15: 1cse_I 1acb_I
16: 2fi4_I 1d0d_B
17: 1r0r_I 1sgp_I

S9.2. Specific Partner Specific Scores (SPIS) and Other Partners Interface Scores (OPIS).

Figure S9.2.1 shows how SIPIS and OPIS scores are collected from each group of equivalent proteins that interact with several distinct partners.

A_1 B_1 C_1 D_1 E_1 F_1 G_1 H_1 I_1 J_1
A_2 B_2 C_2 D_2 E_2 F_2 G_2 H_2 I_2 J_2
A_3 B_3 C_3 D_3 E_3 F_3 G_3 H_3 I_3 J_3
A_4 B_4 C_4 D_4 E_4 F_4 G_4 H_4 I_4 J_4

OPIS	SPIS
s(A_1) s(F_1) s(G_1) s(I_1) s(J_1) s(A_2) s(B_2) s(C_2) s(D_2) s(I_2) s(J_2) s(A_3) s(B_3) s(C_3) s(D_3) s(E_3) s(F_3) s(G_3) s(C_4) s(D_4) s(E_4) s(F_4) s(G_4) s(I_4) s(J_4)	s(B_1) s(C_1) s(D_1) s(E_1) s(E_2) s(F_2) s(G_2) s(I_3) s(J_3) s(A_4) s(B_4)

Figure S9.2.1 Collection of scores for distribution comparison. In this example, there is one group of equivalent proteins whose aligned sequence is ABCDEFGHIJ. Each of these equivalent proteins interacts with a different partner, binding sites are highlighted in yellow, blue, green and red for proteins 1,2,3 and 4, respectively. Scores of specific interface residues are considered for Specific Partner Interface Score (SPIS) distribution. On the other hand, scores for residues that do not belong to its particular interface but they align to residues that belong to the interface of other group members are included in Other Partners Interface Scores (OPIS). Thus, for instance, from protein 1, scores of residues A_1, F_1, G_1, I_1, and J_1 are included in OPIS and scores from residues B_1, C_1, D_1 and E_1 are included in SPIS. Score of residue X of protein N is denoted as s(X_N).

S9.3. Box plots of Specific Partner Specific Scores (SPIS) and Other Partners Interface Scores (OPIS) distributions

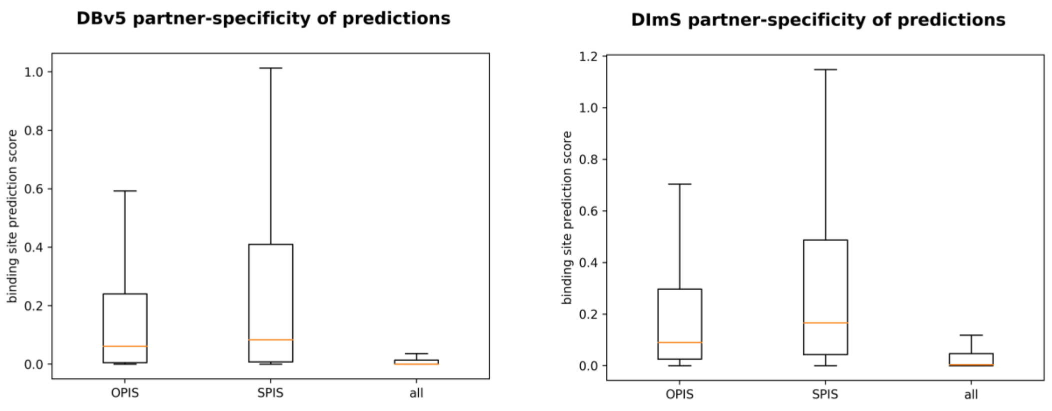

Figure S9.3.1. Distribution of Specific Partner Interface Scores (SPIS) and Other Partners Interface Scores (OPIS). Left, distributions for DBv5; right, distributions for DImS dataset.

S10. Use case PCKS9-PCKS9 & PCKS9-Adnectin

S10.1. Predictions happen to be spatially close to protein active site.

We have employed 3DBIONOTES https://3dbionotes.cnb.csic.es to locate the active site of protein PCKS9. In Figure S10.1.1, residues that belong to the active site are coloured in orange. Prediction of binding sites obtained by BIPSPI are spatially close to the active site. These results are remarkable because Adnectin is known to bind to PCKS9 near the active site (Mitchell *et al.*, 2014) and also PCKS9 self-association is known to happen at the active site (Fan *et al.*, 2008).

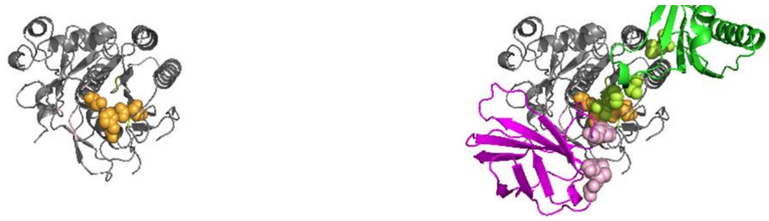

Figure S10.1.1. BIPSPI interface residue predictions for the PCKS9-PCKS9 interaction and for the PCKS9-Adnectin. Chain D (green) and chain E (grey) are PCKS9. Chain G (magenta) is PCSK9-binding adnectin. Just top four highest score predictions are displayed for each interface prediction. Active site is displayed in orange spheres.

S10.2. Comparison with non-partner-specific methods.

We have run SPPIDER (Porollo and Meller, 2007) and ISPRED4 (Savojardo *et al.*, 2017), two non-partner-specific binding site predictors, on the chain B of the pdb 4ov6 in order to compare its predictions with results obtained with BIPSPI. Figure S10.2.1 shows how BIPSPI predictions exhibit partner-specificity, heat map prediction scores displayed in A, B figure-sections are different, and higher scored residues are in contact with their associated partners. On the contrary, predictions obtained with ISPRED4 and SPPIDER (C and D figure-sections) cover both interfaces. Finally, it is worth noting that BIPSIPI tends to predict fewer residues as being part of the interface (most of the residues of the protein are assigned low scores, approximately two order of magnitude smaller than the maximum scores) that other methods. This behaviour is caused by the proposed scoring function (see Main Text Section 2.5) that over represents the residues whose pairs are at the beginning of the pairs prediction list. As a result, smaller but more precise patches are expected as predictions.

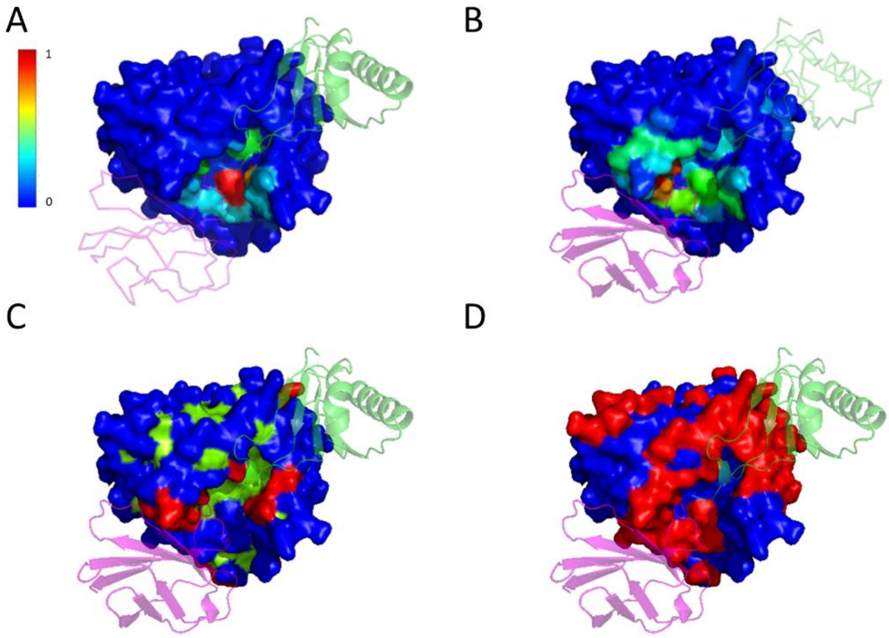

Figure S10.2.1. (A) BIPSPI interface predictions for the interface of PCKS9 chain E and PCKS9 chain D (green ribbon). Normalized (blue minimum, red maximum) scores for all residues are displayed. (B) BIPSPI interface predictions for the interface of PCKS9 chain E and adnectin chain G (magenta ribbon). Normalized (blue minimum, red maximum) scores for all residues are displayed. (C) ISPRED4 binding site predictions for the protein PCKS9 chain E. Red, residue predicted as binding site (score above its recommended threshold); green, non-accessible residue; blue, accessible but not binding site. (D) SPPIDER binding site predictions for the protein PCKS9 chain E. Red, residue-predicted as binding site (score above its recommended threshold); blue, residue predicted as not binding site.

S11. Use case SHR-JACKDAW & SHR-SCR

In this section, we provide another example of how BIPSPI can drive to partner-specific binding site predictions even when those are not fully accurate. PDB entry 5b3h contains the structure of the hetero-complex that is constituted by proteins JACKDAW/IDD10, SHORT-ROOT (SHR) and SCARECROW (SCR). This complex is involved in the regulation of asymmetric cell division and patterning of the root cell types in *Arabidopsis* (Hirano *et al.*, 2017). Specifically, SHR and SCR are responsible for the transcriptional control of the process and to effectively function, they need the cooperation of BIRD/INDETERMINATE DOMAIN (IDD) transcription factors, being JACKDAW/IDD10 one of such proteins (Hirano *et al.*, 2017).

Figure S11.1 displays BIPSPI predictions for the interface of the SHR-JACKDAWN and SHR-SCR interactions. In this case, some of the residues at the interface of SHR-SCR were successfully located, achieving high precision (e.g. 100% for the first 4 residues). BIPSPI was also able to identify residues at the interface of SHR-JACKDAWN, and although the partner-specificity in this example is not perfect, it is still not negligible, as the native binding site residue predicted values have a higher average score than the second predicted patch. Moreover, the precision obtained for these predictions was also high, 75% for the first four residues.

As it was done in Section S10.2, we have also run SPPIDER and ISPRED4 on the same proteins (see Figure S11.2). Again, both SPPIDER and ISPRED4 predictions cover the binding site of both interfaces. As a consequence, the precision of their results is lower than the ones obtained by BIPSPI. This is especially true for the SHR-SC interface and, although BIPSPI predictions for the SHR-JACKDAWN interface are not fully perfect, the precision achieved by BIPSPI is higher than the obtained with other methods.

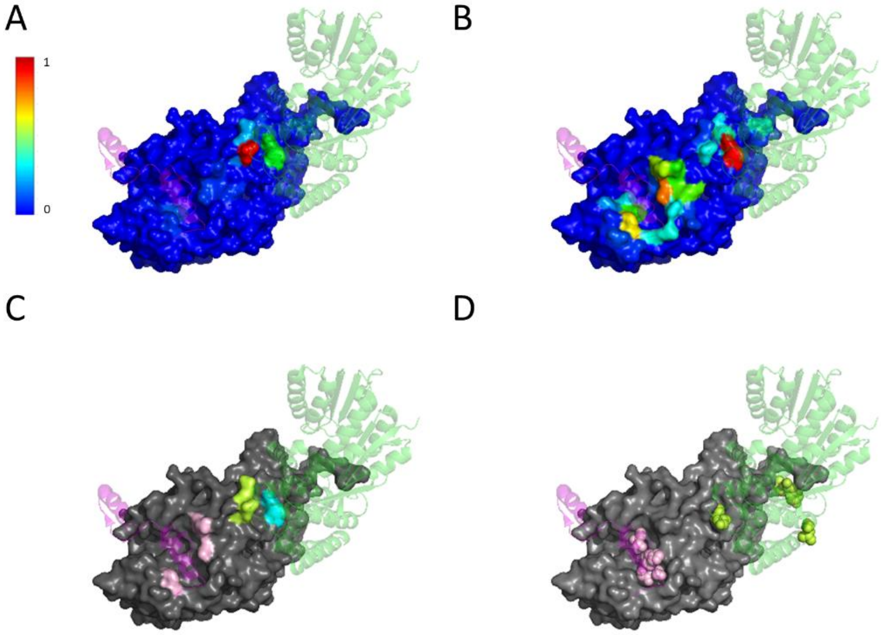

Figure S11.1. BIPSPI interface predictions for the proteins included in pdb 5b3h bioassembly number 1. Chain A (green) is SCR, chain B (grey/heat map) is SHR and Chain C (magenta) is JACKDAW. **(A)** Predictions for SHR (chain B) interface with partner SCR

(chain A) Normalized (blue minimum, red maximum) scores for all residues are displayed. **(B)** Normalized scores (minimum blue, maximum red) for SHR (chain B) interface with partner JACKDAW (chain C). Normalized (blue minimum, red maximum) scores for all residues are displayed. **(C)** Top four highest score predictions for SHR-SCR interaction are coloured as green lemon and cyan. Cyan residue was predicted as binding site for both interfaces, being in this case a true positive. The top four highest score predictions for the SHR-JACKDAW interface are coloured in light pink and cyan. For this interface, the cyan residue represents a false positive. **(D)** Top four highest score predictions of JACKDAW (chain C) interface with SHR (chain B) are shown as light pink spheres and the top four highest score prediction of interface of SCR (chain A) with partner SHR (chain B) are displayed as green lemon spheres.

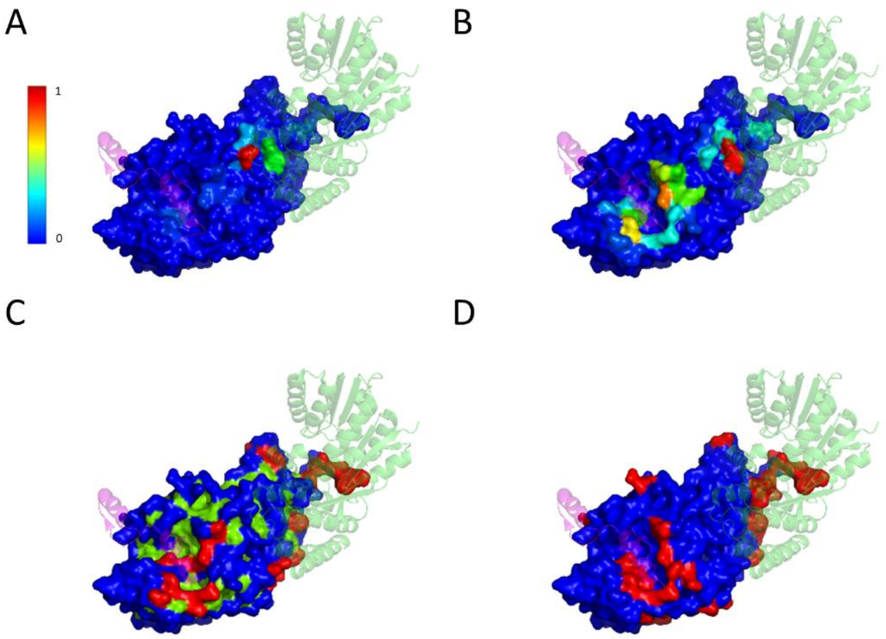

Figure S11.2. (A) BIPSPI interface predictions for the interface of SHR (chain B) and SCR (chain A). Normalized (blue minimum, red maximum) scores for all residues are displayed. **(B)** BIPSPI interface predictions for the interface of SHR (chain B) and JACKDAW (chain C). Normalized (blue minimum, red maximum) scores for all residues are displayed. **(C)** ISPRED4 binding site predictions for the protein SHR (chain B). Red, residue-predicted as binding site (score above its recommended threshold); green, non-accessible residue; blue, accessible but not binding site. **(D)** SPPIDER binding site predictions for the protein SHR (chain B). Red, residue-predicted as binding site (score above its recommended threshold);; blue, residue predicted as not binding site.

S12. Results per complex

Table S12.1 DBv5 complexes and its performance results at complex level using structure-based model. Precision and recall calculated at threshold that maximizes MCC.

**PDB**	ROC-auc RRCP	ROC-auc BS	MCC BS	Precision BS	Recall BS
1A2K	0.9721	0.9112	0.4813	0.3617	0.8293
1ACB	0.9867	0.952	0.6268	0.5714	0.8727
1AHW	0.9819	0.8828	0.4784	0.4273	0.7015
1AK4	0.9099	0.8189	0.3161	0.2449	0.75
1AKJ	0.8597	0.6219	0.1611	0.2651	0.2821

1ATN	0.6852	0.5485	0.0483	0.1209	0.2
1AVX	0.8767	0.8051	0.2568	0.3418	0.4426
1AY7	0.9761	0.9173	0.5431	0.5	0.9048
1AZS	0.9868	0.8882	0.517	0.425	0.7556
1B6C	0.956	0.8926	0.4212	0.4286	0.6
1BGX	0.8734	0.5503	0.0752	0.25	0.1141
1BJ1	0.9752	0.8966	0.4582	0.3824	0.7091
1BKD	0.9485	0.8383	0.3416	0.4778	0.4175
1BUH	0.954	0.8957	0.4877	0.3974	0.7949
1BVK	0.9832	0.9305	0.5448	0.4535	0.8478
1BVN	0.9756	0.9501	0.6216	0.6494	0.6944
1CGI	0.9695	0.9399	0.6706	0.6747	0.8358
1CLV	0.9535	0.9724	0.7655	0.7273	0.875
1D6R	0.9677	0.923	0.6258	0.6026	0.8393
1DE4	0.8856	0.6617	0.1272	0.1461	0.194
1DFJ	0.8986	0.7457	0.2288	0.3626	0.3438
1DQJ	0.9714	0.9066	0.4662	0.4578	0.623
1E4K	0.8961	0.7269	0.3039	0.2935	0.5094
1E6E	0.9369	0.7823	0.3881	0.4	0.5263
1E6J	0.9801	0.9547	0.5093	0.3721	0.8421
1E96	0.8844	0.8018	0.4198	0.375	0.675
1EAW	0.9686	0.9211	0.6049	0.6456	0.7612
1EER	0.9517	0.9168	0.5796	0.7284	0.5728
1EFN	0.8645	0.8451	0.3809	0.3165	0.9259
1EWY	0.8612	0.83	0.2959	0.321	0.52
1EXB	0.9174	0.6287	0.1614	0.2245	0.2037
1EZU	0.9297	0.8258	0.5047	0.6154	0.5581
1F34	0.746	0.6239	0.1242	0.2778	0.3049
1F51	0.9475	0.858	0.3969	0.4935	0.5
1F6M	0.9171	0.793	0.319	0.3736	0.5231
1FAK	0.734	0.5296	-0.0905	0.0395	0.0732
1FC2	0.8885	0.8718	0.4455	0.3827	0.8158
1FCC	0.863	0.7879	0.2976	0.2658	0.5385
1FFW	0.8651	0.7642	0.3515	0.3553	0.75
1FLE	0.9601	0.9277	0.5935	0.561	0.8364
1FQ1	0.9754	0.9171	0.4879	0.4217	0.7292
1FQJ	0.9785	0.9449	0.625	0.5833	0.7925
1FSK	0.9741	0.9014	0.5197	0.4706	0.7018
1GCQ	0.634	0.6395	0.1579	0.3371	0.8333
1GHQ	0.7321	0.5303	0.0869	0.099	0.3846
1GL1	0.9736	0.9745	0.7258	0.6582	0.9455
1GLA	0.8727	0.7574	0.3512	0.3026	0.5476
1GP2	0.9849	0.937	0.5441	0.4524	0.76
1GPW	0.9373	0.7855	0.2698	0.3659	0.4225
1GRN	0.9553	0.8802	0.5318	0.55	0.6984

1GXD	0.8191	0.7687	0.205	0.2043	0.3654
1H1V	0.8676	0.6937	0.2469	0.2989	0.3714
1H9D	0.8455	0.7485	0.3314	0.4941	0.6087
1HCF	0.9262	0.8713	0.4263	0.3537	0.7838
1HE1	0.933	0.869	0.5348	0.5584	0.7288
1HE8	0.9124	0.6978	0.2097	0.1791	0.3529
1HIA	0.9685	0.9485	0.6416	0.6351	0.8246
1I2M	0.9606	0.9103	0.5361	0.6092	0.6092
1I4D	0.8231	0.6027	0.0728	0.1515	0.2632
1I9R	0.9735	0.8458	0.2984	0.2812	0.4576
1IB1	0.9253	0.796	0.2768	0.303	0.4545
1IBR	0.8714	0.7323	0.2494	0.4302	0.3217
1IJK	0.8638	0.6941	0.1841	0.2118	0.4091
1IQD	0.9884	0.9501	0.6103	0.5976	0.7313
1IRA	0.7552	0.6633	0.2516	0.4494	0.3774
1J2J	0.9617	0.9209	0.5511	0.4429	0.9394
1JIW	0.9499	0.9028	0.4971	0.407	0.7447
1JK9	0.9752	0.9315	0.5901	0.5584	0.7818
1JMO	0.949	0.8719	0.4104	0.3933	0.5738
1JPS	0.9783	0.8998	0.4979	0.4731	0.6667
1JTD	0.9731	0.8924	0.5628	0.5408	0.7361
1JTG	0.9391	0.8289	0.4322	0.5455	0.5517
1JWH	0.953	0.8211	0.3538	0.3012	0.5556
1JZD	0.9007	0.8082	0.5975	0.6197	0.6769
1K4C	0.9809	0.9547	0.6186	0.4884	0.8936
1K5D	0.9758	0.8895	0.5277	0.5556	0.6395
1K74	0.9603	0.869	0.4979	0.5125	0.6308
1KAC	0.7108	0.6381	0.1219	0.24	0.3673
1KKL	0.9137	0.6984	0.1776	0.1895	0.4
1KLU	0.9094	0.8317	0.2629	0.2208	0.4857
1KTZ	0.9425	0.8544	0.3986	0.321	0.8966
1KXP	0.9227	0.7208	0.1925	0.3218	0.2642
1KXQ	0.9266	0.8345	0.3842	0.4545	0.4667
1LFD	0.9298	0.8829	0.4921	0.3836	0.875
1M10	0.8104	0.6459	0.1695	0.2812	0.3649
1M27	0.8443	0.7495	0.371	0.2593	0.9545
1MAH	0.9529	0.8951	0.4785	0.5067	0.5758
1ML0	0.8408	0.8391	0.3537	0.3474	0.5
1MLC	0.9532	0.8712	0.2796	0.2692	0.4667
1MQ8	0.9463	0.8529	0.4542	0.3953	0.7556
1N2C	0.9862	0.8244	0.3716	0.466	0.3453
1NCA	0.9742	0.8465	0.3164	0.3297	0.4412
1NSN	0.975	0.9116	0.5236	0.4554	0.7727
1NW9	0.8386	0.7345	0.1557	0.2527	0.4423
1OC0	0.68	0.6713	0.182	0.2024	0.4359

1OFU	0.9168	0.7516	0.1919	0.2069	0.3913
1OPH	0.9917	0.9428	0.537	0.3951	0.8421
1OYV	0.8884	0.7919	0.4291	0.5616	0.5256
1PPE	0.9786	0.9816	0.7682	0.7089	0.9655
1PVH	0.9815	0.9307	0.4504	0.3095	0.8667
1PXV	0.7879	0.7273	0.3644	0.5062	0.5694
1QA9	0.7378	0.6539	0.1808	0.3077	0.5854
1QFW	0.8882	0.8169	0.2551	0.3194	0.4182
1R0R	0.985	0.9737	0.6928	0.5949	0.94
1R6Q	0.671	0.5947	0.0039	0.2338	0.3396
1R8S	0.9578	0.9122	0.5982	0.6795	0.6974
1RKE	0.8051	0.6892	0.2684	0.3908	0.4416
1RLB	0.888	0.724	0.2419	0.2234	0.4565
1RV6	0.9035	0.8154	0.3645	0.4	0.6538
1S1Q	0.8767	0.8679	0.45	0.4268	0.8333
1SBB	0.9416	0.8199	0.323	0.25	0.6389
1SYX	0.8021	0.6492	0.163	0.2812	0.6585
1T6B	0.8274	0.7529	0.2977	0.3034	0.4286
1TMQ	0.9746	0.934	0.5735	0.5854	0.6761
1UDI	0.9728	0.9541	0.736	0.7342	0.8657
1US7	0.7831	0.6554	0.0732	0.1667	0.3721
1VFB	0.9851	0.9242	0.5062	0.4419	0.7917
1WDW	0.9845	0.9193	0.5347	0.5814	0.5682
1WEJ	0.9597	0.8909	0.3723	0.3196	0.6327
1WQ1	0.9521	0.9114	0.6396	0.7532	0.6517
1XD3	0.9763	0.938	0.5754	0.4861	0.875
1XQS	0.829	0.7384	0.21	0.3333	0.375
1XU1	0.8619	0.8278	0.3566	0.3529	0.5769
1Y64	0.8821	0.6353	0.1514	0.2474	0.2637
1YVB	0.9711	0.9322	0.5495	0.4505	0.8723
1Z0K	0.9463	0.9307	0.5206	0.4267	0.9143
1Z5Y	0.9682	0.9205	0.6558	0.5833	0.913
1ZHH	0.9025	0.7615	0.1869	0.2174	0.3846
1ZHI	0.8732	0.7111	0.2161	0.2609	0.5455
1ZLI	0.7504	0.8132	0.449	0.4951	0.7286
1ZM4	0.9013	0.7832	0.3143	0.2784	0.4737
2A1A	0.9626	0.8854	0.4223	0.325	0.7429
2A5T	0.897	0.783	0.2651	0.3182	0.4242
2A9K	0.7985	0.6833	0.1532	0.2603	0.3455
2ABZ	0.965	0.9317	0.567	0.5	0.8125
2AJF	0.8163	0.6178	0.0627	0.1235	0.1724
2AYO	0.8697	0.8069	0.3624	0.4756	0.5
2B42	0.9127	0.7688	0.4215	0.5222	0.5165
2B4J	0.8583	0.6296	0.2076	0.2169	0.5
2BTF	0.8823	0.7957	0.2855	0.3412	0.4531

2C0L	0.7916	0.7408	0.2526	0.3662	0.3939
2CFH	0.9453	0.8953	0.5845	0.6622	0.7101
2FD6	0.9777	0.9013	0.3495	0.2581	0.6316
2FJU	0.9309	0.6041	0.0918	0.0989	0.2368
2G77	0.9793	0.9463	0.6775	0.7037	0.76
2GAF	0.7566	0.5639	0.02	0.1625	0.1262
2GTP	0.9873	0.9598	0.7222	0.6522	0.8824
2H7V	0.9603	0.8504	0.2697	0.2551	0.5556
2HLE	0.9063	0.8731	0.497	0.5844	0.6429
2HMI	0.9893	0.8841	0.3133	0.2283	0.5122
2HQS	0.9327	0.8078	0.4143	0.5063	0.5063
2HRK	0.9708	0.9324	0.5986	0.5185	0.8936
2I25	0.9008	0.7526	0.239	0.3222	0.6042
2I9B	0.89	0.6938	0.247	0.4091	0.4286
2IDO	0.7518	0.7241	0.2359	0.3218	0.5957
2J0T	0.8824	0.8991	0.5351	0.506	0.8077
2J7P	0.9821	0.9301	0.6085	0.6413	0.7024
2JEL	0.9329	0.9112	0.4038	0.3929	0.5893
2MTA	0.953	0.8492	0.2953	0.3188	0.4231
2NZ8	0.9584	0.8823	0.5093	0.6	0.5926
2O3B	0.9196	0.7975	0.3374	0.3617	0.5965
2O8V	0.9532	0.8668	0.3319	0.2152	0.7727
2OOB	0.6104	0.7156	0.2762	0.2821	0.9565
2OOR	0.9682	0.886	0.4303	0.4078	0.5753
2OT3	0.962	0.9047	0.5747	0.6234	0.6857
2OUL	0.9844	0.9584	0.7007	0.7403	0.7917
2OZA	0.8733	0.7835	0.3641	0.4138	0.4865
2PCC	0.9287	0.8282	0.3166	0.2683	0.6111
2SIC	0.9841	0.9428	0.563	0.5	0.8182
2SNI	0.9912	0.9738	0.7223	0.6711	0.8947
2UUY	0.9814	0.9248	0.5558	0.5195	0.8163
2VDB	0.7745	0.7476	0.2165	0.2258	0.4038
2VIS	0.9856	0.9068	0.4275	0.3488	0.6522
2VXT	0.9758	0.9056	0.5211	0.4842	0.7077
2W9E	0.9612	0.913	0.5188	0.4526	0.7544
2X9A	0.5562	0.4718	-0.0379	0.3171	0.52
2YVJ	0.9373	0.8186	0.3371	0.321	0.5417
2Z0E	0.9389	0.8706	0.4437	0.5055	0.6053
3A4S	0.9363	0.7842	0.2797	0.2708	0.7647
3AAA	0.8852	0.8364	0.4112	0.3976	0.569
3AAD	0.7707	0.6918	0.1927	0.25	0.4231
3BIW	0.8082	0.621	0.1367	0.15	0.2857
3BP8	0.6752	0.4709	0.1108	0.1282	0.2381
3BX7	0.8405	0.7882	0.4602	0.5542	0.6571
3CPH	0.9785	0.943	0.5669	0.4588	0.8125

3D5S	0.6335	0.6364	0.1493	0.2235	0.4043
3DAW	0.9688	0.9216	0.58	0.6765	0.6053
3EO1	0.9768	0.8729	0.4531	0.3654	0.717
3EOA	0.9723	0.9104	0.4133	0.3649	0.6
3F1P	0.8728	0.7772	0.3567	0.4607	0.6949
3FN1	0.6836	0.5828	0.1251	0.3226	0.4762
3G6D	0.9777	0.9441	0.5464	0.4667	0.7778
3H11	0.9627	0.9384	0.6205	0.6914	0.6914
3H2V	0.7543	0.6001	-0.0044	0.1461	0.3939
3HI6	0.9776	0.9229	0.5386	0.4889	0.7213
3HMX	0.9814	0.8562	0.4358	0.4022	0.5873
3K75	0.7977	0.6554	0.0464	0.1429	0.2667
3L5W	0.9887	0.9634	0.5601	0.3696	0.9714
3L89	0.8237	0.6377	0.1259	0.2062	0.2703
3LVK	0.9209	0.8507	0.3016	0.2588	0.4783
3MXW	0.9776	0.898	0.421	0.3846	0.625
3P57	0.9112	0.8561	0.3496	0.3239	0.6571
3PC8	0.9408	0.8646	0.3866	0.3704	0.8571
3R9A	0.9455	0.7921	0.2363	0.2258	0.3684
3RVW	0.9841	0.9256	0.4942	0.3864	0.7556
3S9D	0.9197	0.7803	0.3491	0.4024	0.6
3SGQ	0.9569	0.9369	0.574	0.4615	0.9474
3SZK	0.8213	0.5982	0.0829	0.16	0.2791
3V6Z	0.961	0.873	0.3791	0.3673	0.5806
3VLB	0.9542	0.8506	0.4524	0.4945	0.5625
4CPA	0.9785	0.9389	0.5084	0.4125	0.825
4DN4	0.9864	0.965	0.5414	0.3656	0.9444
4FQI	0.9534	0.5922	0.1541	0.134	0.2549
4FZA	0.8759	0.64	0.3053	0.3171	0.4727
4G6J	0.9719	0.887	0.4097	0.4157	0.5692
4G6M	0.9613	0.8865	0.3369	0.3333	0.5179
4GAM	0.8651	0.8076	0.3688	0.5851	0.2806
4GXU	0.9687	0.6859	0.1459	0.1707	0.1918
4H03	0.8832	0.5509	0.1447	0.1842	0.2545
4HX3	0.7687	0.5971	0.1725	0.32	0.3582
4IZ7	0.8316	0.775	0.3029	0.2474	0.6316
4JCV	0.9459	0.8434	0.1939	0.2151	0.3077
4LW4	0.9838	0.9434	0.5097	0.4375	0.6731
4M76	0.806	0.6101	0.1621	0.1683	0.4359
7CEI	0.9153	0.7729	0.2917	0.3587	0.7021
9QFW	0.9441	0.8583	0.3602	0.358	0.58
BAAD	0.6598	0.5539	0.1917	0.2444	0.4314
BOYV	0.8678	0.8174	0.4331	0.382	0.7234
BP57	0.9412	0.8497	0.4042	0.3333	0.75
CP57	0.9285	0.8676	0.3677	0.3023	0.7647

mean	0.9052	0.8135	0.3778	0.3903	0.5958

Table S12.2 DBv5 complexes and its performance results at complex level using sequence-based model. Precision and recall calculated at threshold that maximizes MCC.

PDB	ROC-auc RRCP	ROC-auc BS	MCC BS	Precision BS	Recall BS
1A2K	0.7975	0.7253	0.1471	0.1652	0.4634
1ACB	0.9775	0.9124	0.5312	0.4842	0.8364
1AHW	0.974	0.8892	0.4383	0.4158	0.6269
1AK4	0.7981	0.6791	0.085	0.1389	0.4688
1AKJ	0.753	0.6389	0.2237	0.297	0.3846
1ATN	0.5684	0.5596	0.0882	0.1382	0.3091
1AVX	0.934	0.7803	0.2815	0.3158	0.5902
1AY7	0.8889	0.8036	0.3567	0.3763	0.8333
1AZS	0.7121	0.6332	0.0384	0.1059	0.2
1B6C	0.7562	0.6791	0.1138	0.1897	0.4
1BGX	0.8257	0.5698	0.1147	0.2783	0.1739
1BJ1	0.9715	0.8777	0.3921	0.3095	0.7091
1BKD	0.8388	0.7023	0.2843	0.4167	0.3883
1BUH	0.8289	0.7971	0.3163	0.2737	0.6667
1BVK	0.9908	0.9321	0.5597	0.43	0.9348
1BVN	0.9465	0.9081	0.5645	0.5543	0.7083
1CGI	0.9666	0.907	0.5763	0.5729	0.8209
1CLV	0.9396	0.9468	0.6992	0.6753	0.8125
1D6R	0.9604	0.9008	0.5135	0.4796	0.8393
1DE4	0.5251	0.3449	-0.0428	0.0092	0.0149
1DFJ	0.8013	0.6493	0.2224	0.3364	0.3854
1DQJ	0.9754	0.9143	0.5282	0.4646	0.7541
1E4K	0.7319	0.7216	0.2722	0.2596	0.5094
1E6E	0.7996	0.6885	0.1495	0.2021	0.3333
1E6J	0.9695	0.9381	0.5359	0.36	0.9474
1E96	0.7913	0.6632	0.2397	0.2232	0.625
1EAW	0.9441	0.9049	0.4693	0.5161	0.7164
1EER	0.8211	0.7558	0.2901	0.4035	0.4466
1EFN	0.8129	0.7706	0.2731	0.2368	1
1EWY	0.7325	0.7322	0.2207	0.2455	0.54
1EXB	0.7104	0.473	-0.0307	0.037	0.037
1EZU	0.8695	0.6718	0.2609	0.3243	0.5581
1F34	0.6679	0.6165	0.151	0.2797	0.4024
1F51	0.7809	0.7338	0.3052	0.3977	0.4605
1F6M	0.845	0.6657	0.1472	0.2526	0.3692
1FAK	0.6315	0.5148	-0.0348	0.0784	0.1951
1FC2	0.8182	0.8189	0.3652	0.3295	0.7632
1FCC	0.7831	0.7675	0.3409	0.2596	0.6923
1FFW	0.5723	0.6546	0.1958	0.2667	0.6667

1FLE	0.8372	0.8344	0.4151	0.4333	0.7091
1FQ1	0.8522	0.7684	0.2546	0.2353	0.5833
1FQJ	0.9445	0.8542	0.5566	0.4941	0.7925
1FSK	0.9618	0.8655	0.4071	0.3421	0.6842
1GCQ	0.4972	0.4274	-0.183	0.2549	0.7222
1GHQ	0.7214	0.6123	0.0922	0.102	0.3846
1GL1	0.956	0.9451	0.6923	0.6296	0.9273
1GLA	0.4073	0.5827	0.199	0.1868	0.4048
1GP2	0.9397	0.844	0.4259	0.3273	0.72
1GPW	0.7569	0.6479	0.1345	0.2556	0.3239
1GRN	0.8934	0.8096	0.4267	0.4421	0.6667
1GXD	0.5345	0.623	0.0902	0.1215	0.25
1H1V	0.8357	0.6319	0.0453	0.1339	0.2143
1H9D	0.6969	0.63	0.2342	0.4035	0.6667
1HCF	0.6706	0.7042	0.2046	0.2124	0.6486
1HE1	0.9281	0.8366	0.4325	0.4327	0.7627
1HE8	0.9053	0.6881	0.2031	0.1714	0.3529
1HIA	0.9118	0.8644	0.5055	0.5	0.7895
1I2M	0.8751	0.7707	0.3724	0.4312	0.5402
1I4D	0.8968	0.6375	0.1252	0.191	0.2982
1I9R	0.9778	0.8707	0.3769	0.3404	0.5424
1IB1	0.7218	0.7228	0.299	0.3036	0.5152
1IBR	0.6817	0.5346	0.0876	0.2558	0.287
1IJK	0.6863	0.5798	0.0205	0.11	0.25
1IQD	0.9629	0.8558	0.4775	0.4224	0.7313
1IRA	0.6908	0.636	0.1152	0.3186	0.3396
1J2J	0.9289	0.8966	0.4869	0.3721	0.9697
1JIW	0.9139	0.8626	0.3865	0.3	0.7021
1JK9	0.7248	0.6411	0.1723	0.25	0.4545
1JMO	0.82	0.7176	0.2359	0.2393	0.459
1JPS	0.9666	0.9044	0.4356	0.4019	0.6515
1JTD	0.935	0.8992	0.5187	0.5	0.7083
1JTG	0.839	0.747	0.2604	0.3818	0.4828
1JWH	0.4135	0.494	0.0004	0.065	0.1778
1JZD	0.8917	0.7542	0.5013	0.5	0.6462
1K4C	0.9573	0.9346	0.5141	0.3846	0.8511
1K5D	0.8914	0.7827	0.3939	0.4132	0.5814
1K74	0.7928	0.7083	0.2283	0.2598	0.5077
1KAC	0.6982	0.6789	0.2022	0.2589	0.5918
1KKL	0.7524	0.6353	0.1644	0.1828	0.3778
1KLU	0.6622	0.6381	0.0623	0.0917	0.2857
1KTZ	0.7392	0.6649	0.1659	0.2017	0.8276
1KXP	0.8503	0.6689	0.1337	0.2353	0.3019
1KXQ	0.8749	0.8675	0.4546	0.4519	0.6267
1LFD	0.8749	0.8013	0.3314	0.2653	0.8125

1M10	0.6614	0.6009	0.0909	0.2143	0.3649
1M27	0.7733	0.6359	0.1342	0.1607	0.8182
1MAH	0.8521	0.8158	0.3481	0.3252	0.6061
1ML0	0.5458	0.6406	0.1692	0.1982	0.3333
1MLC	0.9689	0.895	0.4009	0.3261	0.6667
1MQ8	0.8725	0.7282	0.1762	0.2119	0.5556
1N2C	0.7722	0.6433	0.1573	0.2264	0.1727
1NCA	0.9682	0.796	0.3557	0.3037	0.6029
1NSN	0.9602	0.8742	0.4552	0.4343	0.6515
1NW9	0.74	0.7985	0.3256	0.34	0.6538
1OC0	0.6443	0.6584	0.17	0.1786	0.5128
1OFU	0.5705	0.4999	-0.0315	0.0667	0.1522
1OPH	0.969	0.8645	0.3512	0.2302	0.7632
1OYV	0.8675	0.8326	0.4088	0.4717	0.641
1PPE	0.9413	0.9523	0.796	0.7308	0.9828
1PVH	0.8671	0.7741	0.2369	0.1835	0.6667
1PXV	0.6569	0.6445	0.1898	0.3406	0.6528
1QA9	0.694	0.6238	0.1621	0.2889	0.6341
1QFW	0.8488	0.7744	0.294	0.3125	0.5455
1R0R	0.9759	0.9531	0.5704	0.4943	0.86
1R6Q	0.6713	0.5898	0.168	0.3187	0.5472
1R8S	0.9022	0.8566	0.5221	0.5745	0.7105
1RKE	0.6212	0.4682	-0.0664	0.1558	0.3117
1RLB	0.7585	0.6769	0.1134	0.1376	0.3261
1RV6	0.6735	0.591	0.1359	0.2577	0.4808
1S1Q	0.7057	0.6244	0.0911	0.2368	0.6429
1SBB	0.8658	0.761	0.2313	0.1905	0.5556
1SYX	0.7437	0.6654	0.2116	0.2925	0.7561
1T6B	0.7602	0.688	0.156	0.1863	0.3016
1TMQ	0.9481	0.8986	0.5083	0.44	0.7746
1UDI	0.7666	0.7547	0.3206	0.4059	0.6119
1US7	0.4387	0.4931	0.0416	0.1481	0.3721
1VFB	0.9868	0.9184	0.5063	0.4141	0.8542
1WDW	0.7951	0.5726	0.0491	0.1287	0.1477
1WEJ	0.9354	0.8715	0.4021	0.313	0.7347
1WQ1	0.8966	0.8066	0.4955	0.5745	0.6067
1XD3	0.8426	0.7314	0.2277	0.2476	0.65
1XQS	0.7082	0.6319	0.0812	0.2255	0.3194
1XU1	0.7116	0.774	0.2942	0.3011	0.5385
1Y64	0.6764	0.5095	0.033	0.1429	0.1978
1YVB	0.9107	0.8224	0.42	0.3254	0.8723
1Z0K	0.8448	0.8258	0.3997	0.3333	0.8857
1Z5Y	0.8826	0.8315	0.4139	0.3956	0.7826
1ZHH	0.6783	0.6329	0.085	0.1406	0.3462
1ZHI	0.6491	0.5655	0.0426	0.1639	0.4545

1ZLI	0.8303	0.8521	0.5205	0.5179	0.8286
1ZM4	0.7375	0.7644	0.2133	0.1885	0.4035
2A1A	0.6823	0.6441	0.1105	0.1364	0.4286
2A5T	0.6979	0.588	0.0856	0.1776	0.2879
2A9K	0.6848	0.5977	0.0908	0.1983	0.4182
2ABZ	0.9249	0.9027	0.4921	0.4149	0.8125
2AJF	0.7245	0.6825	0.2137	0.2105	0.4138
2AYO	0.7106	0.6309	0.1732	0.3187	0.3718
2B42	0.8026	0.6902	0.2061	0.3241	0.3846
2B4J	0.5442	0.5753	0.1378	0.16	0.5556
2BTF	0.754	0.7395	0.2673	0.2835	0.5625
2C0L	0.8	0.7016	0.2039	0.2857	0.4848
2CFH	0.5676	0.5469	0.0015	0.2362	0.4348
2FD6	0.9691	0.8384	0.2706	0.1964	0.5789
2FJU	0.9305	0.6637	0.1272	0.1196	0.2895
2G77	0.8738	0.8352	0.4	0.4369	0.6
2GAF	0.5779	0.4356	-0.0324	0.1181	0.1456
2GTP	0.9381	0.9018	0.5603	0.4938	0.7843
2H7V	0.9477	0.789	0.2452	0.2281	0.5778
2HLE	0.6006	0.4847	-0.0353	0.2	0.3143
2HMI	0.9647	0.8517	0.3047	0.2095	0.5366
2HQS	0.8638	0.7603	0.3429	0.4059	0.519
2HRK	0.8541	0.7877	0.347	0.3303	0.766
2I25	0.8504	0.7589	0.3121	0.369	0.6458
2I9B	0.6583	0.5867	0.0403	0.25	0.3095
2IDO	0.5012	0.4758	-0.0768	0.1651	0.383
2J0T	0.531	0.6068	0.113	0.2427	0.4808
2J7P	0.8606	0.7735	0.3744	0.4516	0.5
2JEL	0.9596	0.9312	0.5783	0.4706	0.8571
2MTA	0.7087	0.6577	0.2316	0.2308	0.4615
2NZ8	0.9411	0.8546	0.4839	0.5248	0.6543
2O3B	0.8368	0.7625	0.3042	0.3186	0.6316
2O8V	0.8631	0.7688	0.2077	0.1505	0.6364
2OOB	0.655	0.6567	0.1312	0.241	0.8696
2OOR	0.9144	0.8293	0.3038	0.3056	0.4521
2OT3	0.8218	0.7201	0.2551	0.3333	0.5286
2OUL	0.9112	0.8667	0.5354	0.5273	0.8056
2OZA	0.6692	0.5876	0.1704	0.2619	0.2973
2PCC	0.5831	0.5663	0.0633	0.1176	0.3889
2SIC	0.9792	0.8855	0.4402	0.3839	0.7818
2SNI	0.9859	0.9379	0.5913	0.5393	0.8421
2UUY	0.9484	0.8704	0.4345	0.413	0.7755
2VDB	0.6768	0.7926	0.2824	0.2626	0.5
2VIS	0.9793	0.8926	0.3993	0.2833	0.7391
2VXT	0.9561	0.8639	0.4422	0.375	0.7385

2W9E	0.9434	0.8823	0.4509	0.3818	0.7368
2X9A	0.4656	0.4909	-0.0407	0.3204	0.66
2YVJ	0.8372	0.787	0.2461	0.24	0.5
2Z0E	0.709	0.5623	0.0528	0.2143	0.3158
3A4S	0.7804	0.7306	0.2372	0.2553	0.7059
3AAA	0.8363	0.7542	0.27	0.2613	0.5
3AAD	0.5854	0.5213	0.1391	0.2159	0.3654
3BIW	0.6987	0.6052	0.1092	0.1167	0.3333
3BP8	0.5703	0.4519	0.0208	0.0648	0.1667
3BX7	0.7496	0.6803	0.242	0.3707	0.6143
3CPH	0.8827	0.8203	0.3231	0.2812	0.5625
3D5S	0.5736	0.6369	0.1337	0.2111	0.4043
3DAW	0.8579	0.7713	0.344	0.3853	0.5526
3EO1	0.9587	0.8281	0.3146	0.265	0.5849
3EOA	0.974	0.914	0.4145	0.3452	0.6444
3F1P	0.5545	0.4896	-0.0297	0.2566	0.4915
3FN1	0.6035	0.5418	-0.0134	0.2455	0.4286
3G6D	0.9272	0.9012	0.4164	0.3486	0.7037
3H11	0.7319	0.6522	0.1105	0.254	0.3951
3H2V	0.6671	0.6243	0.1129	0.1905	0.6061
3HI6	0.986	0.9422	0.6125	0.5204	0.8361
3HMX	0.9637	0.7639	0.1943	0.2054	0.3651
3K75	0.5548	0.4651	-0.0594	0.0855	0.2222
3L5W	0.99	0.9666	0.5196	0.3182	1
3L89	0.6492	0.58	0.1046	0.1881	0.2568
3LVK	0.7342	0.7086	0.2175	0.1881	0.413
3MXW	0.9757	0.8957	0.5051	0.3932	0.8214
3P57	0.9401	0.8886	0.5024	0.3465	1
3PC8	0.7039	0.5539	0.1045	0.2385	0.7429
3R9A	0.7988	0.7099	0.1929	0.1818	0.3509
3RVW	0.9763	0.8534	0.3339	0.2522	0.6444
3S9D	0.7537	0.6382	0.1537	0.2647	0.4909
3SGQ	0.9471	0.8746	0.4148	0.3302	0.9211
3SZK	0.6265	0.5334	0.0066	0.11	0.2558
3V6Z	0.9593	0.8724	0.4283	0.36	0.7258
3VLB	0.8206	0.6892	0.2625	0.3083	0.4625
4CPA	0.9788	0.9674	0.6482	0.4878	1
4DN4	0.9846	0.9599	0.573	0.3889	0.9722
4FQI	0.9176	0.5018	0.0721	0.0741	0.1569
4FZA	0.6723	0.6582	0.1323	0.1692	0.4
4G6J	0.9555	0.8853	0.4227	0.3925	0.6462
4G6M	0.9667	0.8838	0.33	0.31	0.5536
4GAM	0.6938	0.7061	0.312	0.4771	0.2653
4GXU	0.9481	0.5684	0.0606	0.0874	0.1233
4H03	0.6806	0.4639	0.0217	0.0847	0.1818

4HX3	0.6868	0.6387	0.2	0.3163	0.4627
4IZ7	0.7748	0.6932	0.1253	0.1466	0.4474
4JCV	0.8666	0.7582	0.1689	0.176	0.3385
4LW4	0.8006	0.7023	0.2234	0.2135	0.3654
4M76	0.8591	0.7038	0.2305	0.2083	0.5128
7CEI	0.5649	0.4796	-0.0031	0.2185	0.5532
9QFW	0.9453	0.8773	0.3808	0.3556	0.64
BAAD	0.6517	0.5582	0.15	0.2095	0.4314
BOYV	0.9054	0.8682	0.3615	0.3333	0.6596
BP57	0.9193	0.8593	0.3931	0.2935	0.8438
CP57	0.9135	0.8637	0.3753	0.3	0.7941
mean	0.8022	0.7286	0.2647	0.292	0.5532

Table S12.3. DImS complexes and its performance results at complex level using structure-based model. Precision and recall calculated at threshold that maximizes MCC.

PDB	ROC-auc RRCP	ROC-auc BS	MCC BS	Precision BS	Recall BS
1acb	0.9899	0.9726	0.7163	0.6235	0.9636
1avw	0.8855	0.7686	0.2706	0.369	0.4559
1ay7	0.9717	0.9129	0.5756	0.5063	0.9524
1b41	0.889	0.8149	0.3206	0.3077	0.5424
1b6c	0.9572	0.8985	0.5452	0.4842	0.7931
1bdj	0.4095	0.4609	0.03	0.0976	0.381
1blx	0.6886	0.64	0.2124	0.2762	0.4462
1bun	0.4786	0.1878	-0.2567	0.0562	0.1852
1c1y	0.9646	0.9022	0.4843	0.4048	0.8718
1cse	0.9889	0.975	0.6976	0.5882	0.9615
1cxz	0.596	0.5348	-0.0158	0.1889	0.3208
1d0d	0.8531	0.6509	0.0715	0.022	1
1dev	0.6821	0.573	0.1568	0.4545	0.4762
1ds6	0.9124	0.7922	0.3923	0.4681	0.6027
1dtd	0.9527	0.911	0.4981	0.4396	0.7843
1e44	0.839	0.7703	0.3573	0.5854	0.6761
1e96	0.8982	0.802	0.2903	0.2697	0.6
1eai	0.9446	0.8597	0.4782	0.49	0.7656
1em8	0.8831	0.6972	0.2589	0.3214	0.587
1euv	0.8972	0.8526	0.4283	0.5238	0.6286
1f2s	0.9723	0.9701	0.7127	0.6386	0.9636
1f34	0.834	0.7637	0.3368	0.5	0.4563
1fm0	0.9388	0.8521	0.4336	0.5	0.7705
1g0v	0.8893	0.9373	0.6741	0.8243	0.6778
1gl1	0.9663	0.958	0.6973	0.622	0.9444
1gzs	0.944	0.9169	0.6436	0.7473	0.7312
1h4l	0.9684	0.9426	0.6881	0.7283	0.7791

1he1	0.9545	0.8894	0.5279	0.5376	0.7692
1i1q	0.9663	0.8646	0.4192	0.4796	0.5165
1izn	0.8863	0.8035	0.4582	0.8586	0.4048
1jdh	0.8564	0.8745	0.4432	0.5952	0.4854
1jsd	0.8952	0.777	0.5253	0.8182	0.4898
1jtd	0.9467	0.865	0.4993	0.49	0.6806
1k90	0.7405	0.6429	0.1773	0.4468	0.2561
1ka9	0.9501	0.7752	0.2376	0.4	0.4
1l4d	0.7614	0.645	0.0543	0.1789	0.3208
1luj	0.8883	0.8793	0.4788	0.5556	0.5789
1m9x	0.9205	0.7587	0.2705	0.268	0.6341
1nbf	0.8566	0.8394	0.5306	0.7097	0.5841
1nf3	0.9264	0.7882	0.3595	0.4444	0.597
1oph	0.9946	0.9352	0.4992	0.4138	0.7347
1p57	0.7838	0.6355	0.1268	0.2841	0.3571
1p6a	0.6978	0.6149	0.0631	0.1978	0.36
1pzl	0.8634	0.8308	0.3214	0.275	0.7333
1r0r	0.9843	0.9794	0.686	0.5632	0.98
1r8s	0.976	0.9427	0.6719	0.7262	0.7722
1s1q	0.9054	0.8766	0.4925	0.4239	0.9286
1s6v	0.9282	0.8029	0.266	0.1852	0.625
1s70	0.8852	0.7165	0.1958	0.45	0.2903
1sgp	0.9606	0.9343	0.5425	0.4556	0.9318
1spb	0.9166	0.8602	0.5418	0.6023	0.7067
1sq0	0.9241	0.7815	0.3107	0.3482	0.5571
1sq2	0.8758	0.7728	0.3054	0.3929	0.6226
1stf	0.944	0.8697	0.4647	0.44	0.7857
1t6g	0.8892	0.7141	0.1693	0.2609	0.3288
1ta3	0.8794	0.7099	0.3281	0.3804	0.473
1tbg	0.6497	0.5414	0.0178	0.3684	0.2448
1te1	0.8148	0.5881	0.1968	0.2796	0.3939
1tgs	0.9813	0.9698	0.7682	0.7125	0.95
1tx4	0.9504	0.8687	0.5606	0.6024	0.7042
1u0s	0.89	0.8026	0.3529	0.4	0.7391
1uad	0.8638	0.7982	0.3586	0.3258	0.7436
1ugh	0.9396	0.9191	0.5732	0.5957	0.7887
1uuz	0.9056	0.8263	0.4405	0.4583	0.7719
1uw4	0.8391	0.7067	0.1593	0.2929	0.4394
1v74	0.9456	0.9282	0.6565	0.6087	0.9825
1wmi	0.5181	0.5324	0.1204	0.7426	0.7143
1wpx	0.9693	0.9067	0.545	0.617	0.6105
1wrd	0.8883	0.7828	0.4066	0.37	0.9487
1wui	0.8967	0.8557	0.4437	0.7596	0.3835
1xd3	0.9793	0.9315	0.6715	0.716	0.7945
1xl3	0.954	0.8875	0.5288	0.4878	0.8163

1y4h	0.9212	0.8615	0.4909	0.5604	0.7183
1y8x	0.8391	0.7689	0.2625	0.3721	0.5818
1yro	0.9795	0.9393	0.4574	0.3125	0.8824
1z3e	0.7557	0.5457	-0.056	0.2083	0.4651
1zc3	0.7937	0.6154	0.0842	0.2593	0.3621
2a5d	0.9426	0.833	0.4169	0.3932	0.7667
2ajf	0.6752	0.6018	-0.0192	0.0619	0.1034
2bex	0.9034	0.7917	0.3272	0.466	0.4324
2ccl	0.834	0.7929	0.4104	0.4891	0.7627
2d5r	0.7938	0.6877	0.1746	0.2442	0.42
2d7c	0.9464	0.9293	0.4978	0.3864	0.9714
2e2d	0.9413	0.8798	0.4769	0.5146	0.7162
2f4m	0.9502	0.9114	0.565	0.4762	0.8511
2f6m	0.7558	0.7046	0.3425	0.6436	0.7558
2f9z	0.9116	0.7491	0.2645	0.3152	0.537
2fi4	0.9711	0.909	0.5267	0.4681	0.8627
2ftx	0.9163	0.8971	0.6221	0.6585	0.9474
2fun	0.9563	0.8362	0.3736	0.3471	0.6462
2g2u	0.954	0.8539	0.4432	0.505	0.622
2g45	0.9611	0.8538	0.3642	0.2979	0.9032
2ga9	0.7113	0.5267	0.0074	0.1495	0.1524
2grn	0.868	0.7944	0.3298	0.2885	0.7317
2gzj	0.8279	0.6713	0.2264	0.3579	0.6415
2hle	0.906	0.7944	0.3067	0.4149	0.5571
2ido	0.858	0.7802	0.4201	0.4752	0.7619
2ie4	0.8023	0.6097	0.1655	0.2083	0.2857
2j7q	0.9594	0.9251	0.5938	0.5341	0.8704
2o3b	0.9739	0.8923	0.5098	0.4434	0.8246
2ot3	0.9798	0.9308	0.6841	0.7179	0.7671
2p49	0.8189	0.7557	0.2317	0.2772	0.6667
2pr3	0.8693	0.7509	0.3294	0.3913	0.6316
2puo	0.8575	0.7796	0.3615	0.4368	0.7755
2qdy	0.8599	0.8578	0.4782	0.9072	0.4513
2r25	0.9721	0.9408	0.5947	0.5679	0.8214
2r2l	0.9496	0.8255	0.432	0.7732	0.3713
2tld	0.8273	0.7168	0.1561	0.1125	0.5294
2uuy	0.9822	0.942	0.5783	0.5119	0.8776
2uyz	0.893	0.7542	0.2318	0.2571	0.7105
2vut	0.5334	0.6247	0.1843	0.2614	0.434
3bx1	0.9289	0.8126	0.4148	0.4239	0.619
3cr3	0.9537	0.8974	0.5018	0.3977	0.875
3d5r	0.8041	0.7228	0.2354	0.2921	0.4906
3fap	0.9201	0.7782	0.3176	0.2766	0.8387
4cpa	0.9864	0.9479	0.5417	0.4268	0.875
4sgb	0.9459	0.8892	0.5048	0.4831	0.8431

mean	0.8789	0.7985	0.3831	0.4438	0.6492

Table S12.4. DImS complexes and its performance results at complex level using sequence-based model. Precision and recall calculated at threshold that maximizes MCC:

PDB	ROC-auc RRCP	ROC-auc BS	MCC BS	Precision BS	Recall BS
1acb	0.9678	0.8986	0.6241	0.6508	0.7455
1avw	0.8679	0.7594	0.3706	0.4211	0.5882
1ay7	0.9117	0.8467	0.406	0.4198	0.8095
1b41	0.6955	0.6456	0.1931	0.2439	0.339
1b6c	0.8035	0.6964	0.2496	0.3125	0.431
1bdj	0.5226	0.5969	0.2162	0.1875	0.5714
1blx	0.641	0.5545	0.0206	0.1558	0.1846
1bun	0.3366	0.2247	-0.2133	0.0753	0.2593
1c1y	0.832	0.7155	0.2001	0.2658	0.5385
1cse	0.9799	0.9677	0.7628	0.7586	0.8462
1cxz	0.593	0.56	0.1195	0.2727	0.3962
1d0d	0.6726	0.2629	-0.0676	0.0116	0.5
1dev	0.4405	0.4137	-0.0486	0.325	0.3095
1ds6	0.8131	0.7094	0.1802	0.3529	0.3288
1dtd	0.8883	0.8724	0.4507	0.4235	0.7059
1e44	0.6135	0.5978	0.1113	0.4494	0.5634
1e96	0.7315	0.675	0.1586	0.2051	0.4
1eai	0.8563	0.8616	0.5176	0.5152	0.7969
1em8	0.6648	0.5564	-0.0046	0.1765	0.3261
1euv	0.8143	0.7498	0.3478	0.5	0.5
1f2s	0.9018	0.916	0.7103	0.7377	0.8182
1f34	0.685	0.6804	0.2114	0.39	0.3786
1fm0	0.9089	0.877	0.5938	0.7049	0.7049
1g0v	0.6983	0.8216	0.4643	0.5914	0.6111
1gl1	0.9394	0.9402	0.663	0.6522	0.8333
1gzs	0.8764	0.8268	0.4545	0.6375	0.5484
1h4l	0.6805	0.6421	0.1064	0.2892	0.2791
1he1	0.859	0.7715	0.4493	0.5714	0.5538
1i1q	0.8013	0.747	0.2347	0.35	0.3077
1izn	0.6289	0.5792	0.1131	0.519	0.1952
1jdh	0.7973	0.7765	0.4958	0.6711	0.4951
1jsd	0.4569	0.4227	-0.0259	0.2842	0.1837
1jtd	0.9127	0.8361	0.39	0.4211	0.5556
1k90	0.5318	0.5791	0.0806	0.3483	0.189
1ka9	0.7093	0.5539	0.0443	0.2533	0.2
1l4d	0.5808	0.5865	0.0494	0.1786	0.283
1luj	0.8224	0.8352	0.3154	0.4471	0.4
1m9x	0.8028	0.7549	0.284	0.2927	0.5854
1nbf	0.7742	0.6383	0.3252	0.561	0.4071

1nf3	0.7412	0.649	0.3068	0.4217	0.5224
1oph	0.961	0.7373	0.3133	0.2692	0.5714
1p57	0.6869	0.5527	0.0683	0.2442	0.3
1p6a	0.7247	0.6254	0.1538	0.2449	0.48
1pzl	0.5795	0.6727	0.2141	0.2459	0.5
1r0r	0.9662	0.9586	0.6609	0.6308	0.82
1r8s	0.8604	0.8151	0.4367	0.5542	0.5823
1s1q	0.7497	0.6764	0.2153	0.3059	0.619
1s6v	0.8719	0.8171	0.2453	0.1772	0.5833
1s70	0.5422	0.4834	-0.076	0.1765	0.0968
1sgp	0.9722	0.9584	0.7031	0.6452	0.9091
1spb	0.7845	0.7491	0.4661	0.5679	0.6133
1sq0	0.7491	0.6005	0.0748	0.2069	0.2571
1sq2	0.7689	0.7538	0.3335	0.4177	0.6226
1stf	0.8524	0.7968	0.3357	0.4	0.5714
1t6g	0.8123	0.7178	0.2543	0.3373	0.3836
1ta3	0.7336	0.6116	0.2402	0.3118	0.3919
1tbg	0.5208	0.5861	0.2246	0.5568	0.3427
1te1	0.512	0.5377	0.0757	0.1977	0.2576
1tgs	0.9473	0.9052	0.5633	0.6143	0.7167
1tx4	0.8631	0.7836	0.4084	0.5135	0.5352
1u0s	0.771	0.7662	0.3558	0.4247	0.6739
1uad	0.895	0.8267	0.4062	0.3784	0.7179
1ugh	0.8208	0.7651	0.2536	0.4125	0.4648
1uuz	0.7188	0.6167	0.0733	0.2651	0.386
1uw4	0.762	0.6758	0.2135	0.3506	0.4091
1v74	0.8219	0.7594	0.3176	0.4684	0.6491
1wmi	0.5134	0.4985	-0.0217	0.6944	0.4762
1wpx	0.8016	0.634	0.1337	0.2738	0.2421
1wrd	0.6312	0.5513	0.0849	0.2625	0.5385
1wui	0.7123	0.6572	0.1162	0.3978	0.1796
1xd3	0.922	0.8061	0.4279	0.5513	0.589
1xl3	0.4819	0.5332	0.0341	0.1939	0.3878
1y4h	0.7024	0.6217	0.1323	0.3415	0.3944
1y8x	0.6612	0.6321	0.1665	0.3118	0.5273
1yro	0.7287	0.559	0.0231	0.0978	0.2647
1z3e	0.5809	0.4754	-0.0667	0.1974	0.3488
1zc3	0.8377	0.7449	0.2841	0.3947	0.5172
2a5d	0.81	0.7432	0.2741	0.3483	0.5167
2ajf	0.568	0.5904	0.0226	0.092	0.1379
2bex	0.7045	0.686	0.2324	0.3978	0.3333
2ccl	0.4909	0.4764	-0.0214	0.2667	0.339
2d5r	0.7075	0.6927	0.179	0.2647	0.36
2d7c	0.7578	0.8419	0.4099	0.3733	0.8
2e2d	0.8344	0.7968	0.326	0.4432	0.527

2f4m	0.5992	0.6369	0.1548	0.2361	0.3617
2f6m	0.6247	0.5969	0.1279	0.5647	0.5581
2f9z	0.823	0.745	0.2381	0.32	0.4444
2fi4	0.9467	0.8764	0.4486	0.4512	0.7255
2ftx	0.7369	0.6311	0.1843	0.4744	0.6491
2fun	0.7424	0.6498	0.0369	0.15	0.1846
2g2u	0.8089	0.6793	0.1273	0.2907	0.3049
2g45	0.9249	0.7491	0.3281	0.3151	0.7419
2ga9	0.4802	0.4129	-0.0323	0.1111	0.0857
2grn	0.6062	0.5523	0.0558	0.1609	0.3415
2gzj	0.6076	0.5649	0.0911	0.2976	0.4717
2hle	0.7084	0.5386	0.0416	0.25	0.2571
2ido	0.6387	0.571	0.0445	0.2809	0.3968
2ie4	0.6609	0.5317	-0.0377	0.0488	0.0571
2j7q	0.9213	0.8573	0.5131	0.5441	0.6852
2o3b	0.8849	0.7974	0.3963	0.425	0.5965
2ot3	0.892	0.8188	0.4031	0.4937	0.5342
2p49	0.7199	0.6101	0.0497	0.1978	0.4286
2pr3	0.6491	0.6234	0.1031	0.2703	0.3509
2puo	0.6785	0.6807	0.2432	0.3924	0.6327
2qdy	0.505	0.4981	-0.0679	0.4194	0.2
2r25	0.8156	0.7844	0.3122	0.4118	0.5
2r2l	0.7346	0.6635	0.229	0.5769	0.2228
2tld	0.9887	0.959	0.3957	0.2	1
2uuy	0.9498	0.8791	0.5009	0.5	0.7347
2uyz	0.677	0.5387	0.0574	0.1923	0.3947
2vut	0.6276	0.6368	0.2278	0.3026	0.434
3bx1	0.8407	0.6851	0.1593	0.2529	0.3492
3cr3	0.9141	0.8156	0.3632	0.3467	0.65
3d5r	0.6164	0.5277	0.0963	0.2043	0.3585
3fap	0.75	0.6715	0.1931	0.2466	0.5806
4cpa	0.9531	0.9475	0.621	0.481	0.95
4sgb	0.9127	0.9066	0.5912	0.5833	0.8235
mean	0.7469	0.6883	0.2421	0.3663	0.4798

Table S12.5 CAPRI targets used for independent validation and its performance results at complex level. Precision and recall calculated at threshold that maximizes MCC.

Target	PDB	Used in	ROC-auc interacting pairs BIPSPI	ROC-Auc interacting pairs PAIRpred	ROC-Auc binding site	MCC	Precision	Recall
T58	4G9S	Ispred4 & PAIRpred	0.927	0.897	0.822	0.389	0.375	0.943
T56	4EEF	Ispred4 & PAIRpred	0.796	0.763	0.701	0.000	0.097	0.887
T40	3E8L	Ispred4 & PAIRpred	0.907	0.921	0.767	0.243	0.212	0.951
T39	3FM8	Ispred4 & PAIRpred	0.839	0.796	0.702	-0.050	0.000	0.000

T32	3BX1	Ispred4 & PAIRpred	0.882	0.897	0.710	0.194	0.157	0.800
T41	2WPT	Ispred4 & PAIRpred	0.938	0.858	0.902	0.634	0.846	0.611
T50	3R2X	Ispred4 & PAIRpred	0.831	0.903	0.679	0.058	0.058	1.000
T47	3U43	Ispred4 & PAIRpred	0.954	0.889	0.857	0.452	0.400	0.875
T29	2VDU	Ispred4 & PAIRpred	0.965	0.829	0.874	0.202	0.133	1.000
T01	1KKL	Ispred4	0.875	-	0.638	0.107	0.106	1.000
T03	1KEN	Ispred4	0.964	-	0.691	0.013	0.049	0.838
T08	1NPE	Ispred4	0.820	-	0.628	-0.08544	0.058824	0.0508
T10	1URZ	Ispred4	0.719	-	0.716	0.262	0.479	0.344
T11	1OHZ	Ispred4	0.919	-	0.935	0.697	0.700	0.894
T13	1YNT	Ispred4	0.959	-	0.836	0.310	0.288	0.552
T14	1S70	Ispred4	0.891	-	0.654	0.197	0.208	0.980
T15	1V74	Ispred4	0.936	-	0.898	0.360	0.385	1.000
T16	1TA3	Ispred4	0.961	-	0.880	0.245	0.191	1.000
T22	1SYX	Ispred4	0.750	-	0.632	0.020	0.238	0.122
T23	2B8W	Ispred4	0.896	-	0.779	0.260	0.2920	0.904
T25	2J59	Ispred4	0.947	-	0.868	0.108	0.169	1.000
T27	2O25	Ispred4	0.877	-	0.604	0.074	0.076	0.667
T38	3FM8	Ispred4	0.684	-	0.633	0.273	0.370	0.294
T46	3Q87	Ispred4	0.883	-	0.649	0.154	0.095	0.667
T53	4JW2	Ispred4	0.9508	-	0.857	0.294	0.25	1
T54	4JW3	Ispred4	0.9417	-	0.900	0.582	0.631	0.774
MEAN			All: 0.885; used in PAIRpred: 0.927	0.8614	0.762	0.230	0.264	0.7366

Table S12.6 Performance evaluation for CAPRI binding site prediction, all scores mixed together.

Auc binding site	MCC	Precision	Recall
0.763	0.297	0.315	0.549

References

Fan,D. *et al.* (2008) Self-association of human PCSK9 correlates with its LDLR-degrading activity. *Biochemistry*, **47**, 1631–9.

Heffernan,R. *et al.* (2015) Improving prediction of secondary structure, local backbone angles and solvent accessible surface area of proteins by iterative deep learning. *Sci. Rep.*, **5**, 11476.

Hirano,Y. *et al.* (2017) Structure of the SHR-SCR heterodimer bound to the BIRD/IDD transcriptional factor JKD. *Nat. Plants*, **3**, 17010.

Minhas,F. ul A.A. *et al.* (2014) PAIRpred: partner-specific prediction of interacting residues from sequence and structure. *Proteins*, **82**, 1142–55.

Mitchell,T. *et al.* (2014) Pharmacologic Profile of the Adnectin BMS-962476, a Small Protein Biologic Alternative to PCSK9 Antibodies for Low-Density Lipoprotein Lowering. *J. Pharmacol. Exp. Ther.*, **350**, 412–424.

Platt,J.C. and Platt,J.C. (1999) Probabilistic Outputs for Support Vector Machines and Comparisons to Regularized Likelihood Methods. *Adv. LARGE MARGIN Classif.*, 61--74.

Porollo,A. and Meller,J. (2007) Prediction-based fingerprints of protein-protein interactions. In, *Proteins: Structure, Function and Genetics*. Wiley Subscription Services, Inc., A Wiley Company, pp. 630–645.

Savojardo,C. *et al.* (2017) ISPRED4: Interaction sites PREDiction in protein structures with a refining grammar model. *Bioinformatics*, **33**, 1656–1663.

Yuan,Z. (2005) Better prediction of protein contact number using a support vector regression analysis of amino acid sequence. *BMC Bioinformatics*, **6**, 248.

Zadrozny,B. and Elkan,C. (2002) Transforming classifier scores into accurate multiclass probability estimates. In, *Proceedings of the eighth ACM SIGKDD international conference on Knowledge discovery and data mining - KDD '02.*, p. 694.
